# Negative valuation of ambiguous feedback may predict near-term risk for suicide attempt in Veterans at high risk for suicide

**DOI:** 10.3389/fpsyt.2024.1492332

**Published:** 2025-01-30

**Authors:** Catherine E. Myers, Rokas Perskaudas, Vibha Reddy, Chintan V. Dave, John G. Keilp, Arlene King, Kailyn Rodriguez, Lauren St. Hill, Rachael Miller, Alejandro Interian

**Affiliations:** ^1^ Research Service, VA New Jersey Health Care System, Department of Veterans Affairs, East Orange, NJ, United States; ^2^ Department of Pharmacology, Physiology and Neuroscience, New Jersey Medical School, Rutgers University, Newark, NJ, United States; ^3^ Mental Health and Behavioral Services, VA New Jersey Health Care System, Department of Veterans Affairs, Lyons, NJ, United States; ^4^ War Related Illness and Injury Study Center (WRIISC), East Orange, NJ, United States; ^5^ Center for Pharmacoepidemiology and Treatment Science, Institute for Health, Health Care Policy and Aging Research, Rutgers University, New Brunswick, NJ, United States; ^6^ Department of Psychiatry, Columbia University College of Physicians and Surgeons, New York State Psychiatric Institute, New York, NY, United States; ^7^ Department of Psychology, Rutgers University School of Arts and Sciences, Piscataway, NJ, United States; ^8^ Department of Psychiatry, Robert Wood Johnson Medical School, Rutgers University, Piscataway, NJ, United States

**Keywords:** suicide, feedback learning, reinforcement learning, computational model, software

## Abstract

**Background:**

Learning from feedback – adapting behavior based on reinforcing and punishing outcomes – has been implicated in numerous psychiatric disorders, including substance misuse, post-traumatic stress disorder, and depression; an emerging literature suggests it may also play a role in suicidality. This study examined whether a feedback-based learning task with rewarding, punishing and ambiguous outcomes, followed by computational modeling, could improve near-term prospective prediction of suicide attempt in a high-risk sample.

**Method:**

Veterans (N=60) at high-risk for suicide were tested on a task of reward- and punishment-based learning, at multiple sessions across a one-year period. Each session was coded according to whether the participant had (1) an actual suicide attempt (ASA); (2) another suicide-related event (OtherSE) such as suicidal behavior or suicidal ideation-related hospital admission (but not an ASA); or (3) neither (noSE) in the next 90 days. Computational modeling was used to estimate latent cognitive variables including learning rates from positive and negative outcomes, and the subjective value of ambiguous feedback.

**Results:**

Optimal responding on the reward-based trials was positively associated with upcoming ASA, and remained predictive even after controlling for other standard clinical variables such as current suicidal ideation severity and prior suicide attempts. Computational modeling revealed that patients with upcoming ASA tended to view ambiguous outcomes as similar to weak punishment, while OtherSE and noSE both tended to view the ambiguous outcome as similar to weak reward. Differences in the reinforcement value of the neutral outcome remained predictive for ASA even after controlling for current suicidal ideation and prior suicide attempts.

**Conclusion:**

A reinforcement learning task with ambiguous neutral outcomes may provide a useful tool to help predict near-term risk of ASA in at-risk patients. While most individuals interpret ambiguous feedback as mildly reinforcing (a “glass half full” interpretation), those with upcoming ASA tend to view it as mildly punishing (a “glass half empty” interpretation). While the current results are based on a very small sample with relatively few ASA events, and require replication in a larger sample, they provide support for the role of negative biases in feedback-based learning in the cognitive profile of suicide risk.

## 1 Introduction

Death by suicide is a public health epidemic across demographic and geographic groups, with increasing rates among younger adults as well as Veterans and active military members (1), despite considerable investments in research, policy, and clinical programs (2). Although there has been intensive study of epidemiological risk factors for suicide (3), an emerging priority is the identification not only of which individuals are at risk, but also when that risk is highest (4), so that clinical and supportive interventions can be applied in a timely and effective manner. For this reason, recent studies have begun to consider near-term factors that indicate upcoming risk within a more acute time window of days to weeks (5), as well as examining cognitive changes that underlie an individual’s transition from suicidal ideation to actual suicide attempt (6).

One important aspect of cognition is feedback-based learning. Individuals adjust their behavior by learning from reinforcing and punishing outcomes, in order to adapt to daily life circumstances (7). Altered feedback-based learning has been implicated in a number of psychiatric disorders including addiction (8), post-traumatic stress disorder (9), and anxiety and depression (10), and may also be a pathway contributing to suicidal behavior. In one prior study, older adult participants learned responses through trial-and-error, with variable reward rates and contingencies; results suggested that that depressed individuals with history of suicide attempt showed a tendency to discount prior feedback in favor of more recent feedback (11). Such learning biases could be linked to suicidality by leading individuals to discount historical experiences, and instead focus on more recent negative events in guiding their current behavior. Findings such as these are consistent with the role of positive valence systems (including reward responsiveness, reward learning, and reward valuation) in suicide-related psychopathology (12). Feedback-based learning tasks therefore represent a potential inroad to understanding the cognitive changes that underlie risk for suicide.

Feedback-based learning tasks also lend themselves to computational modeling, which can provide an explanatory link from behavior to the psychobiological mechanisms underlying those observable behaviors (13, 14). Reinforcement-learning (RL) models learn through trial-and-error to adapt their behavior in order to maximize rewards and avoid punishments. Importantly, the key teaching signal in RL models – prediction error – has been associated with mesolimbic dopamine systems (15), linking the latent cognitive variables extracted by RL models with the brain substrates of learning (16). Blunting of prediction error has been observed in depressed patients with a history of suicide attempt, and is correlated with blunted value signals in the ventromedial prefrontal cortex (17).

Applied to individual-level data, RL models can be used to discover a set of model parameters that together best reproduce each participant’s trial-by-trial learning behavior; the parameters correspond to latent cognitive variables such as learning rate to better-than-expected and worse-than-expected outcomes, tendency to exploit previously-successful strategies versus occasionally explore new ones, and the subjective value of reinforcement (18). These latent cognitive variables may be altered in neurological and psychiatric patient groups, thus identifying potential mechanisms that could be driving group differences in behavior (19). For example, one study reported that depressed individuals with a history of suicide attempt had increased learning rate on trials with worse-than-expected outcomes, but only after negative mood induction (20).

However, many existing feedback-based learning tasks conflate learning to obtain reward with learning to avoid punishment, despite the fact that these appear to be distinct systems that can be differentially affected in psychopathology. To dissociate reward-based and punishment-based learning, the probabilistic reward- and punishment-based learning task (RPLT) interleaves reward-based training, which provides positively-valenced feedback for correct responses and no feedback for incorrect responses, with punishment training, which provides negatively-valenced feedback for incorrect responses and no feedback for correct responses (21–24). This allows for separate consideration of reward-based learning and punishment-based learning within a single participant. Thus, for example, the RPLT has been used to show selective impairment on reward-based learning but relative sparing of punishment-based learning in unmedicated patients with depressive disorders (25, 26); unmedicated patients with Parkinson’s disease (PD) show a similar pattern, but dopaminergic medication reverses this pattern, leading to remediated reward-based learning but impaired punishment-based learning (24).

The RPLT also allows the use of RL modeling to examine the subjective reinforcement value of the no-feedback outcome (*R_0_)*, which is ambiguous as it could represent either a failure to obtain reward or else a successful avoidance of punishment. For example, one prior study with the RPLT found that Veterans with severe post-traumatic stress disorder (PTSD) symptoms showed better reward-based learning (but not punishment-based learning), compared to peers with few or no PTSD symptoms; the PTSD group also tended to value *R_0_* significantly less positively (21), exaggerating the perceived difference between reward and non-reward in the task. Thus, RL modeling provided an explanation for the seemingly paradoxical facilitation of reward-based learning in the PTSD group, but also suggested that negatively-biased subjective valuation of ambiguous or neutral events could contribute to PTSD symptoms.

The current study was an initial evaluation of use of the RPLT to prospectively predict suicide attempt within the subsequent 90 days, in a small group of Veterans at risk for suicide. This study builds on the existing literature in two important ways. First, much of the existing literature relating suicidality and learning has employed retrospective research designs, comparing participants with *vs*. without a history of prior suicide attempt; in some cases, attempts may have occurred years or even decades previously. In addition to focusing on prospective prediction of suicide attempt, the current study builds on suicide risk factor research more broadly by evaluating a much-needed shorter window of suicide risk: 90 days. While this can be challenging given the low base rate of suicide behavior, we pursued this goal by recruiting a sample of Veterans at high-risk for suicide within one year post a suicide-related hospital admission, and by conducting multiple assessments over a one-year follow-up period. Second, the current study builds on previous research evaluating the role of feedback-based learning and suicide risk (11, 17), by utilizing a task that allows for separate evaluation of learning from reward *vs*. punishment, and of the subjective valuation of ambiguous neutral feedback.

Our primary hypothesis was that performance on the RPLT could be used to predict near-term (90-day) suicide attempt, above and beyond standard variables used in suicide risk assessment (i.e., suicidal ideation severity and number of prior suicide attempts). Our secondary hypothesis was that latent cognitive variables, extracted from RL modeling of the behavioral data, such as learning rate from rewarding or punishing outcomes and/or the subjective valuation of neutral feedback, would also predict upcoming suicide attempt, indicating specific cognitive processes altered in at-risk individuals entering a period of heightened risk for suicide attempt.

## 2 Materials and methods

### 2.1 Participants

Participants were N = 60 Veterans receiving care through the VA New Jersey Health Care System (VANJHCS) with a history of acute psychiatry admission due to suicide attempt (SA) or severe suicidal ideation (SI) during the prior year. Other inclusion criteria were at least one of the following: (a) actual, interrupted, or aborted suicide attempt during the prior year, as measured by the Columbia Suicide Severity Rating Scale (C-SSRS) (27); or (b) one or more instances of preparatory behavior in the prior year, defined as actions directed toward a suicide attempt that go beyond ideation, such as assembling a specific method (e.g. buying pills, purchasing a gun) or preparing for one’s death by suicide (e.g. giving things away, writing a farewell note); or (c) clinically-significant SI within the prior week, defined as a score of 4+ on the Beck Scale for Suicidal Ideation (SSI) (28). Participants were screened for cognitive capacity using the Montreal Cognitive Assessment (MoCA) (29) and for history of psychiatric disorders using the Mini International Neuropsychiatric Interview (MINI) (30); however, current or past medical or psychiatric diagnoses did not automatically trigger study exclusion if participants were otherwise capable of participating. Based on these inclusion criteria, a transdiagnostic group of participants were recruited, with substance use, head injury, PTSD, and mood disorders being the most common psychiatric difficulties. Full recruitment and screening details for the study are provided in the **Appendix**.

Participants who met enrollment criteria provided written informed consent; at this point, information from screening (including C-SSRS, SSI, MoCA, MINI) became part of the study data. The study was approved and monitored by the VANJHCS Institutional Review Board (protocol #1577294) and conformed to the Declaration of Helsinki and U.S. Federal policy for the protection of human subjects.

### 2.2 Procedures

At baseline (Session 1), participants completed a clinical interview, several self-report questionnaires, and the reward- and punishment-learning task (RPLT) described further below. The clinical interview included updated C-SSRS and SSI (if screening had not occurred on the same day), brief medical history including history of traumatic brain injury (TBI), using the Brief Traumatic Brain Injury Screen (BTBIS) (31) modified to include injuries sustained outside of as well as during military deployment, and combat exposure, using the Combat Exposure Scale (CES) (32). Self-report questionnaires included the Beck Depression Inventory (BDI-II) (33) to assess severity of depression symptoms in previous two weeks; and the Beck Hopelessness Scale (BHS) (34) to assess hopelessness and negative attitudes about the future during the past week. Full details of data collection methods are provided in the **Appendix**.

Participants also completed the reward- and punishment-learning task (RPLT), described below; most also completed additional questionnaires and neurocognitive tasks not reported here. In most cases, neurocognitive testing occurred either immediately after collection of clinical and self-report data (80.5%) or within 24h (11.9%).

Following baseline testing, participants were followed for one year, with follow-up testing (Sessions 2-5) occurring at approximately 3-month intervals. Follow-up sessions included updated clinical interviews (including C-SSRS and SSI), self-report questionnaires, and neurocognitive tasks. In cases where individuals could not travel to the testing site for follow-up testing (e.g., during the COVID-19 pandemic, Spring 2020-Spring 2021), efforts were made to collect interview and questionnaire data by telephone and/or mail, especially updated C-SSRS and SSI, allowing capture of suicide-related events during the follow-up period.

### 2.3 Reward and punishment-learning task

The RPLT was adapted from previously described methods (21, 22), programmed using PsychoPy (35) and presented on a Dell laptop or similar computer, with the subject seated in a quiet room at a comfortable viewing distance from the computer. The RPLT task software is available at: https://osf.io/p328a/.

In brief, the RPLT is a probabilistic categorization task, in which participants learn through trial-and-error to classify each of four stimuli into categories A and B. On each trial, one of four stimuli (*S1, S2, S3, S4*) appears, and the subject is asked to guess whether the stimulus belongs to category *A* or B, by pressing one of two keyboard keys. The visual images for stimuli *S1-S4* are from the Novel Object and Unusual Name (NOUN) Database, 2^nd^ edition (36), downloaded from http://sussex.ac.uk/wordlab/noun (accessed March 2019); a different set of four images is used at each testing session.

**Figure 1:**
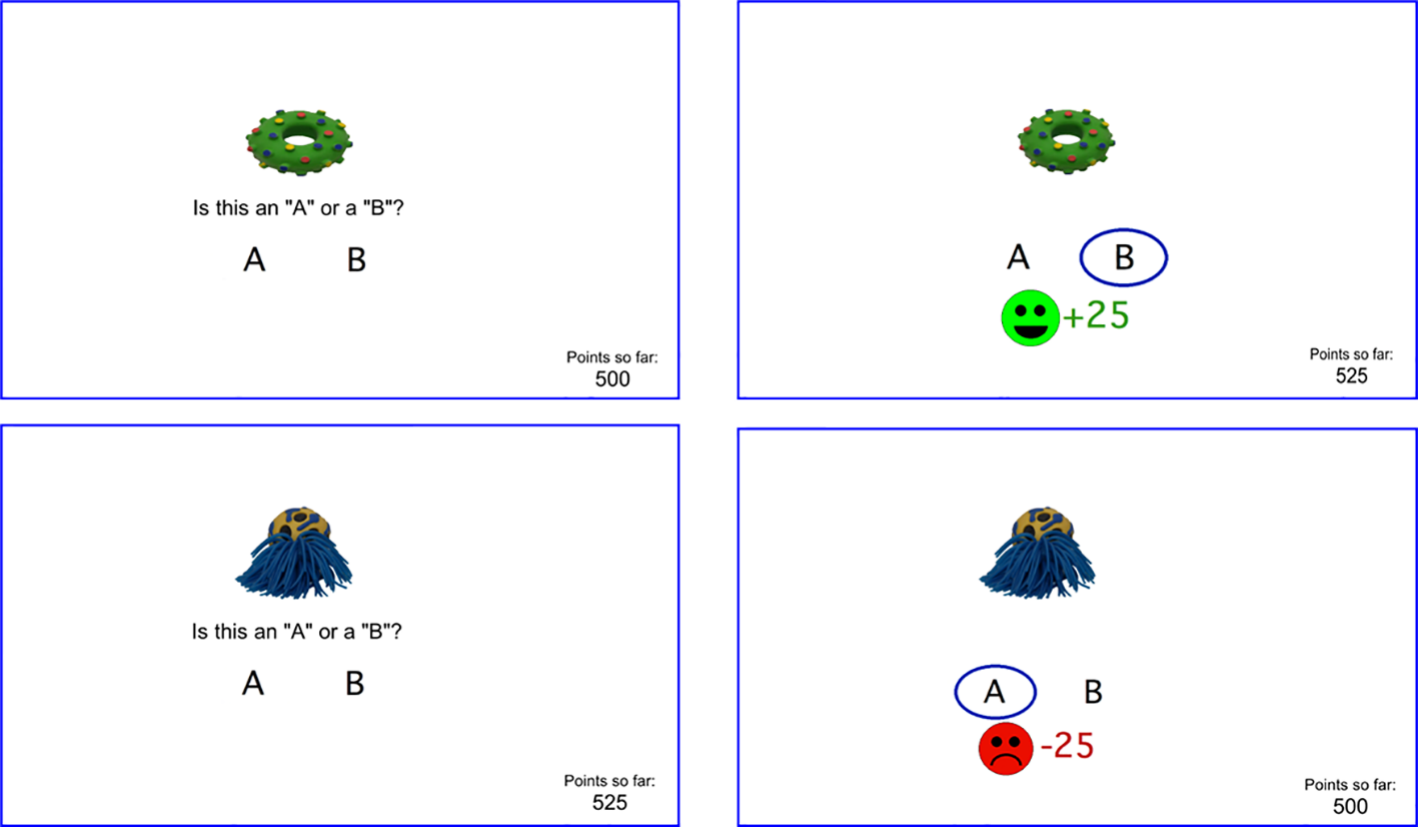
Screenshots from RPLT including (top left) a reward-based trial where the participant responds correctly and wins points (top right), and (bottom left) a punishment-based trial where the participant responds incorrectly and loses points (bottom left). A running point tally appears at lower right of screen. Different stimuli are used at each testing session.

Stimuli *S1* and *S2* are reward-based stimuli, meaning that a correct categorization is rewarded by on-screen feedback (smiley face) and point gain (25 points), while an incorrect categorization receives no feedback (**Figure 1**-top). Stimuli *S3* and *S4* are punishment-based stimuli, meaning that an incorrect categorization is punished by on-screen feedback (frowning face) and point loss (25 points), while correct responses trigger no feedback (**Figure 1**-bottom). Thus, the no-feedback outcome is ambiguous, since on some trials it signals missed opportunity to obtain reward, and on others it signals successful avoidance of punishment. The task thus allows within-subject comparison of learning from reward *vs*. punishment. Participants are not explicitly informed whether each trial is a reward-based or feedback-based trial, and therefore whether the ambiguous (no-feedback) outcome represents a missed reward or a missed loss, although participants can learn across trials which stimuli are associated with potential reward *vs*. potential loss, and therefore infer the meaning of the no-feedback outcome when it occurs.

Category mappings are probabilistic. Stimuli *S1* and *S3* belong to category *A* on 80% of trials and to category *B* on 20% of trials; stimuli *S2* and *S4* belong to category *B* on 80% of trials and category *A* on 20% of trials. The optimal response for each stimulus is therefore the most frequently correct category, regardless of which response is actually correct on a given trial.

Before the first trial, there is a short practice phase (using two stimuli that do not appear during the regular task trials); participants are guided through responding to a reward-based stimulus (once to obtain reward, and once with no feedback) and a punishment-based stimulus (once receiving punishment, and once with no feedback). Participants are instructed to try to maximize point tally and are reminded that the same stimulus does not necessarily belong to the same category every time it appears. They may use either (or both) hands to enter responses.

The task itself comprises 80 trials, including two blocks of 40 trials; each block contains 10 trials with each stimulus, 8 with the more frequent category and 2 with the less frequent category. Trial order within a block is pseudorandom but fixed across subjects, with the constraint that each stimulus is always paired with its more frequently-correct category on the first trial where it appears.

There is no time limit for responding. Responses occurring <200 msec after stimulus onset are discarded as anticipatory responses; the program records the first response after 200 msec, which ends the trial. There is an inter-trial interval of 250 msec, during which the point tally remains visible, before the next stimulus appears. The tally is initialized to 500 points at the start of practice, to ensure the tally does not drop below 0 during the course of the experiment, which could induce subject frustration.

For each trial, the computer records the stimulus, the participant response and whether that was the optimal response (regardless of actual outcome), along with reaction time and any on-screen feedback. Summary behavioral variables analyzed for each subject were percent optimal responses to reward-based and punishment-based stimuli.

### 2.4 Computational modeling

The behavioral data (trial-by-trial stimuli, responses, and feedback) were also used as input to a reinforcement learning (RL) model (7, 18). Following previously-described methods (22, 37), we used a *Q*-learning model (38, 39), which learns an expected value *Q* for each possible response to the current stimulus; on each trial, the model examines the *Q*-values to choose a response, and then updates *Q*-values based on prediction error (*PE*), defined as the difference between the expected outcome and the actual reinforcement received. Model performance is defined by parameters including the learning rate on trials with better-than-expected outcomes (*LR+*), learning rate for trials with worse-than-expected outcomes (*LR-*), the tendency to exploit previously-successful responses *vs*. occasionally explore new ones (*β*), and the subjective value of ambiguous reinforcement (*R_0_*). Individual-level data are simulated by optimizing parameter values such that the model’s trial-by-trial behavior most closely mimics the participant’s trial-by-trial choices; the resulting configuration of parameters represent estimates of latent cognitive variables for that participant. Full details of model building and testing (including predictive check and parameter recovery studies) appear in the **Appendix**.

### 2.5 Prospective outcomes

Following prior methods (40, 41), for each RPLT testing session, we categorized near-term clinical outcomes into one of three mutually exclusive categories, based on previously-published case classification criteria (42): (1) “ASA” if the participant had 1+ actual suicide attempt (ASA), defined as deliberate self-harm with at least some intent of ending one’s life, within the 90-day period following RPLT testing; (2) “OtherSE” if there was no ASA within this window but at least one suicide-related event (SE) which could include interrupted/aborted suicide attempt, defined as acts of deliberate self-harm, with at least some suicidal intent, that are stopped prior to reaching a risk threshold; preparatory behavior such as assembling a method or preparing for one’s death by suicide; or severe suicidal ideation resulting in inpatient or emergency room admission; or (3) “noSE” if there was neither ASA nor other SE within the 90-day follow-up window.

ASAs and other SEs were determined from clinician-administered C-SSRS at all available testing sessions, supplemented by medical chart review. Participants were censored if they died or withdrew from the study (or were withdrawn by the study team, e.g., due to criminal justice involvement) during the 1-year follow-up period; data collected and outcomes occurring prior to censoring remained part of the study record. Participants who were lost to contact during the follow-up period were not considered censored as long as outcomes could be tracked through Veterans Health Administration (VHA) electronic medical records.

### 2.6 Statistical analysis

Generalized estimating equations (GEEs) were used to test the effects of predictors on the multinomial dependent variable. GEEs are an extension of generalized linear models for analysis of repeated measures with non-normal response variables (43–45), and can model longitudinal designs while accounting for correlations between observations within individuals (46–48). Here, the outcome variable had three levels: ASA, OtherSE, or noSE (reference level). Given that the same patient could contribute multiple data points (1-5 observations per subject), we accounted for within-subject clustering. Results are presented by exponentiating the coefficients from the estimated marginal model to obtain odds ratios (OR), with 95% confidence interval (CI); threshold for significance was set at .05.

GEE models were estimated using the *nomLORgee* function for GEE with multinomial outcomes, from the *multgee* package (49) in R v.4.3.1 (50). First, a simple model examined whether outcomes could be predicted by the RPLT behavioral scores (percent optimal responding to reward- and punishment-based stimuli). Second, to test our hypothesis regarding predictive value of behavioral measures, we used a GEE to evaluate the incremental utility of RPLT behavioral scores in predicting the response variable, over standard suicide risk variables (number of lifetime ASAs, SSI at time of testing) (40, 41, 51, 52).

To explore the latent cognitive variables derived from computational modeling, the same methods were used as for the behavioral data, but including the RL parameter estimates for each subject as predictors.

As supplemental analyses, we also examined other pertinent covariates, including PTSD, which has been shown to associate with improved reward-based learning and decreased *R_0_* on this task (21); depression, which has been shown to associate with impaired reward-based learning on this task (25, 26); opioid use; history of traumatic brain injury (TBI); and prior week SE, given that some of our participants were tested while in-patients in the immediate aftermath of an SE, while others were tested after discharge (though within one year of SI-related hospitalization). These supplemental analyses are reported in the **Appendix**.

## 3 Results

### 3.1 Sample characteristics

The sample of 60 Veterans included 7 females (11.7%), and had mean age 45.3 years (SD 13.8, range 23 to 72) at baseline, with average CES score of 9.2 (SD 10.9, range 0 to 39).

A majority of participants (47 of 60) had a prior history of suicide attempt, with n=20 (33.3%) reporting one prior ASA and n=27 (45.0%) reporting multiple (two or more) prior ASAs. Almost all (n=57, 95.0%) endorsed lifetime history of suicidal ideation with some intent, based on C-SSRS ideation severity of 4+, assessed at screening and baseline. About a third (n=22, 36.7%) had lifetime history of non-suicidal self-injurious behavior (NSSI). **Table 1** summarizes other demographic and clinical information for the 60 Veteran participants.

**Table 1:** Demographic and clinical information for 60 Veteran participants at baseline (Session 1).

	*N*	%
Race		
White	36	60.0%
Black or African-American	15	25.0%
Asian	1	1.7%
American Indian	1	1.7%
Other/Mixed	7	11.7%
Ethnicity		
Hispanic/Latino	16	26.7%
Not Hispanic/Latino	44	73.3%
Education		
High school or Graduate Equivalency Degree (GED) or less	23	38.3%
Some college but no degree	24	40.0%
Graduation from 4-year college or higher	13	21.7%
Employment status		
Employed (full-time or part-time)	15	25.0%
Unemployed	40	66.7%
Sheltered workshop (e.g., Clinical Work Therapy program)	1	1.7%
Full-time student	4	6.7%
Marital status		
Married or Living as married	20	33.3%
Never married	15	25.0%
Separated or Divorced	24	40.0%
Widowed	1	1.7%
Psychiatric/Neurological features		
Head Injury/Traumatic Brain Injury (TBI)	41	68.3%
Post-traumatic stress disorder (PTSD)	33	55.0%
Social Phobia*	15	25.9%
Obsessive-Compulsive Disorder (OCD)*	4	6.9%
Generalized Anxiety Disorder (GAD)*	19	32.8%
Major Depressive Disorder (MDD)	27	45.0%
Bipolar Disorder	14	23.3%
Opioid abuse (Lifetime)	19	31.7%
Any substance misuse in past 30 days	51	85.0%
Binge drinking episode in past 30 days	18	30.0%
Baseline (Session 1) Location		
In-patient on acute ward (following SI-related admission)	39	65.0%
Residential Unit	13	21.7%
Outpatient	8	13.3%
Baseline (Session 1): Suicide-related Event (SE) Recency		
Prior Week	30	50.0%
Prior Month (but not Prior Week)	12	20.0%
Prior Year (but not Prior Month)	18	30.0%

Category percentages may not sum to 100% due to rounding error and/or multiple categories per participant. PTSD, social phobia, OCD, GAD, MDD, bipolar based on MINI. *Responses for social phobia, OCD, GAD were not available for 2 subjects; percentages for these disorders are calculated relative to n=58. “Opioid abuse” includes heroin, morphine and prescription painkillers used to get high; “Any substance misuse” includes opioids as well as other drugs (excluding alcohol and tobacco). Suicide-related event (SE) recency calculated based on most recent SE prior to day of RPLT Session 1 testing, or day of Session 1 interviewer assessment for n=2 who did not complete RPLT at Session 1.

During the one-year follow-up period, 4 individuals had one or more ASAs, and 17 had other suicide-related events (OtherSE), including 1 with an interrupted/aborted suicide attempt, 9 with one or more instances of preparatory behavior (but no attempt) and 7 with one or more SI-related hospital admissions (without attempt or preparatory behavior). Five participants were censored, including 2 who died of natural causes (at 205 and 217 days) and 3 withdrawn (1 by request of subject at 77 days, and 2 by study team due to criminal justice involvement at 86 and 125 days). One of the two individuals who died had an ASA at 180 days (more than 90 days after their first and only RPLT testing session); none of the other censored individuals had any suicide-related events before censoring. Data from censored individuals were included in the current analysis as long as censoring occurred >90 days after an RPLT testing session.

Of the 21 participants who had ASAs or other SEs during the one-year follow-up, many had multiple events. In total, there were 7 ASAs, 1 interrupted/aborted attempt, 15 instances of preparatory behavior, and 22 SI-related hospitalizations (excluding hospitalizations that occurred secondary to suicide attempt or preparatory behavior, to avoid double-counting).

### 3.2 RPLT and suicidal outcomes

A total of 144 RPLT sessions were completed. Of these, 21 were excluded because they could not be associated with an outcome due to censoring (study end <90 days after testing, n=16; study withdrawal, n=3; death from natural causes, n=2). An additional 5 files from two participants were dropped due to apparent noncompliance (e.g., participant only pressed one response key during the entire RPLT task). In three additional cases, participants completed only the first 50-63 trials (before computer crash or other interruption), but the available trials were retained and scored.

Thus, 118 files from 58 unique participants were ultimately analyzed. There were no obvious differences in demographics, clinical profile, or outcome distribution among the dropped files compared to the 118 retained for analysis (results not shown). Based on the 90-day window following RPLT testing, 5 were classified as ASA, 13 were classified as OtherSE (including 7 cases with SI-related hospital admissions but no suicidal behavior; 2 with aborted/interrupted suicide attempts, and 4 with preparatory behavior), and the remaining 100 were classified as noSE. (Note that a few additional ASA and OtherSE occurred during the one-year follow-up, but fell outside the 90 day window after an RPLT testing session.) Detailed results for each outcome group are summarized in **Table 2**.

**Table 2:** Behavioral results and RL model variables, by 90-day outcome group.

	NoSE (n=100)		OtherSE (n=13)		ASA (n=5)	
Group Characteristics	N	%	N	%	N	%
Gender=Female	18	18.0%	2	15.4%	0	0.0%
Marital Status=Married or living as married	33	33.0%	5	38.5%	2	40.0%
Major Depression=yes	41	41.0%	6	46.2%	3	60.0%
Bipolar Disorder=yes	26	26%	1	8.3%	2	40.0%
PTSD=yes	61	61.0%	6	46.2%	1	20.0%
History of head injury/TBI=yes	64	64.0%	11	84.6%	0	0.0%
Opioid use (lifetime, at T1)	29	29.0%	4	30.8%	2	40.0%
Group Characteristics	Mean	SD	Mean	SD	Mean	SD
Age, in years (at T1)	45.2	14.2	50.7	11.8	49.8	4.8
Beck Scale for Suicidal Ideation (SSI)	7.3	9.2	6.0	8.6	15.2	8.4
Beck Depression Inventory (BDI-II)	24.2	14.5	20.9	13.2	32.0	4.6
Beck Hopelessness Scale (BHS)	8.3	6.1	5.8	5.0	14.4	7.1
Reward- and Punishment-Learning Task (RPLT)	Mean	SD	Mean	SD	Mean	SD
% Optimal, Reward	52.5	19.5	46.3	12.4	76.0	131
% Optimal, Punishment	59.1	10.3	61.7	10.0	58.0	9.3
% Win-Stay, Reward	67.9	19.9	65.9	21.1	86.5	7.5
% Lose-Shift, Reward	35.1	18.4	35.6	26.9	42.3	15.2
% Win-Stay, Punishment	50.8	12.6	52.4	10.2	54.9	3.7
% Lose-Shift, Punishment	47.8	14.1	53.2	15.6	45.2	4.9
Reinforcement Learning (RL) Model: Parameter Estimates	Mean	SD	Mean	SD	Mean	SD
*LR+*	0.33	0.35	0.24	0.30	0.38	0.39
*LR-*	0.40	0.38	0.37	0.38	0.20	0.17
*β*	0.52	0.29	0.46	0.28	0.32	0.18
*R_0_*	+0.13	0.71	+0.22	0.60	-0.24	0.71
*negLLE*	46.53	9.99	46.99	11.10	42.02	5.67

ASA, actual suicide attempt; OtherSE, other suicide-related event (SE) excluding ASA; noSE, no suicidal event; RPLT % Optimal = percent of trials (with reward-based or punishment-based stimuli) on which participant made optimal (most often correct) categorization response, regardless of actual outcome on that trial; RPLT % Win-Stay = percent of trials (with reward-based or punishment-based stimuli) on which the participant repeated a previously-successful response to the same stimulus; % Lose-Shift = percent of trials on which participant shifted away from a previously-unsuccessful response to that stimulus. Marital status and clinical history were recorded at baseline. RL parameters: LR+/LR- = learning rate on trials with better-than-expected/worse-than-expected outcomes (range 0 to 1); β = explore/exploit parameter (values near 0 indicate tendency to repeat previously-successful responses; values near 1 indicate tendency to explore other responses); R_0_ = subjective value of the ambiguous/neutral outcome (+1 is similar to explicit reward, -1 is similar to explicit punishment, 0 is truly neutral). Note that each unique participant could have contributed more than one datafile to this analysis; within-subject effects are not controlled for in this table, but are included in the GEE models.

### 3.3 RPLT and prediction of suicide attempts

**Figure 2:**
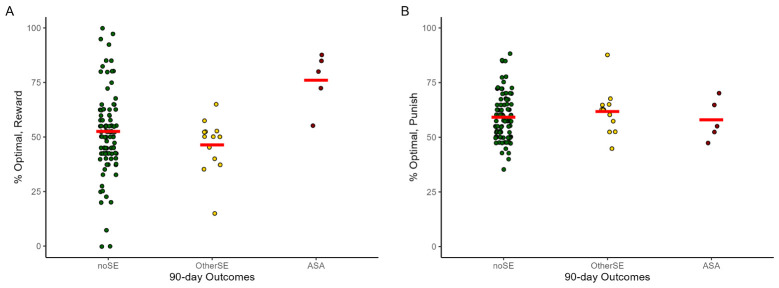
Percent optimal responding to **(A)** reward-based stimuli and **(B)** punishment-based stimuli on the RPLT, across the 118 data files that could be associated with 90-day prospective outcomes. Note each participant could contribute RPLT data from multiple testing sessions. ASA, actual suicide attempt; OtherSE, other suicide-related event, excluding ASA; noSE, no ASA or other SE. Plots created using ggplot2 (59) for R; red lines indicate group mean and dots represent individual data points. The GEE analysis, which takes into account the repeated-measures structure of the data (not depicted in this figure) indicated increased reward-based learning was significantly predictive of upcoming ASA (OR=1.08, 95% CI=1.03-1.12, p<.001) but not OtherSE (OR=0.98, 95%CI=0.96-1.01, p=.138), compared to the noSE reference group.

Compared to the noSE reference group, percent optimal responding on reward-based trials was higher in the ASA group (**Figure 2A**; OR=1.08 [1.03-1.12], p<.001), but not the OtherSE group (OR=0.98 [0.96-1.01], p=.138). Percent optimal responding on punishment-based trials was not different from noSE in either the ASA group (**Figure 2B**; OR=0.95 [0.89-1.01], p=.087) or the OtherSE group (OR=1.02 [0.97-1.08], p=.420).

Our primary hypothesis was that RPLT behavior could predict upcoming ASA, above and beyond the contributions of other standard risk variables. **Table 3** summarizes the results of the GEE model using RPLT variables as predictors, adjusting for suicide risk variables of total lifetime ASAs (assessed at baseline) and SSI score (assessed at time of RPLT testing). Odds of ASA increased with greater number of prior ASAs and higher percent optimal responding on reward-based trials. No parameters emerged as significant predictors of OtherSE.

**Table 3:** Results of GEE model, predicting actual suicide attempt (ASA) or other suicidal event excluding ASA (OtherSE) within 90 days, based on RPLT behavioral variables.

	Actual Suicide Attempt (ASA)			Other Suicidal Event (excluding ASA)		
	OR	95% CI	p-value	OR	95% CI	p-value
RPLT: % optimal, reward-based trials	**1.10**	**1.05-1.16**	**<.001**	0.98	0.96-1.01	.144
RPLT: % optimal, punishment-based trials	0.94	0.84-1.05	.281	1.02	0.97-1.07	.501
SSI at time of RPLT testing	1.12	0.99-1.26	.080	0.98	0.91-1.06	.663
# Prior ASA (lifetime)	**1.64**	**1.10-2.44**	**.015**	0.71	0.45-1.12	.138

GEE model includes session as repeated-measure, adjusted for covariates including severity of suicidal ideation (SSI), number of ASA prior to study entry, presence of PTSD, and SE within the week prior to RPLT testing. OR, odds ratio; CI, confidence interval. Bold indicates predictors for which p<.05.

### 3.4 Reinforcement learning model

**Figure 3:**
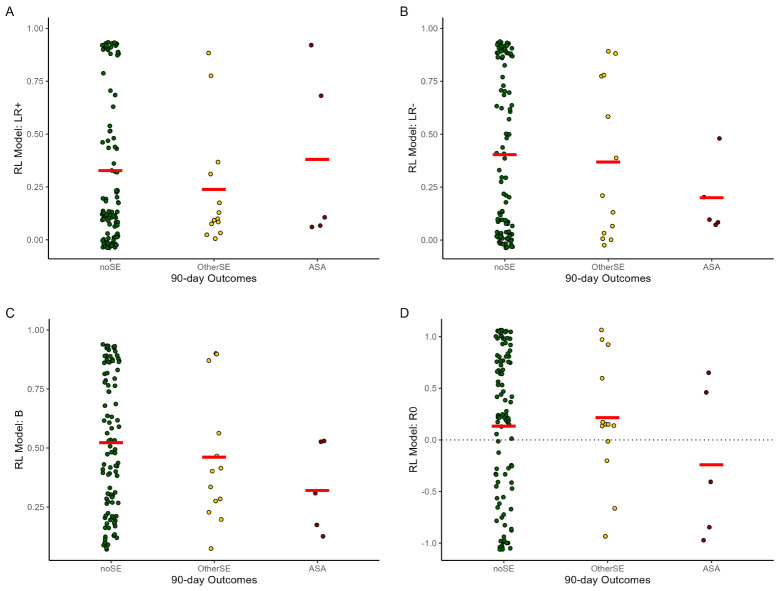
Mean parameter estimates in the reinforcement learning (RL) model, by 90-day outcome group. **(A, B)** LR+/LR- = learning rate on trials with better- or worse-than-expected outcomes; **(C)** *β* = explore/exploit parameter (higher values = more tendency to explore); **(D)** *R_0_* = subjective value of ambiguous/neutral feedback (*R_0_* >0 equivalent to reward; *R_0_* <0 equivalent to punishment); other abbreviations as in **Figure 2**. Note each participant could contribute data from 1+ testing session. Plots created using ggplot2 (59) for R; red lines indicate group mean and dots represent individual data points. The GEE analysis, which takes into account the repeated-measures structure of the data (not depicted in this figure) indicated that lower *R_0_* was significantly predictive of upcoming ASA (OR=0.44, 95% CI=0.22-0.89, p=.022) but not OtherSE (OR=1.42, 95% CI=0.56-3.64, p=.460), compared to the noSE reference group.

The RL model was run on the 118 data files, and parameter estimates derived for each (**Table 2**). As shown in **Figure 3**, whereas the noSE group tended to show *R_0_>*0 (i.e., valuing the neutral/ambiguous outcome as similar to a mild reward), the ASA group tended to show *R_0_* <0 (i.e., valuing the neutral ambiguous outcome as similar to a mild punisher) (OR 0.44 [0.22-0.89], p=.022). Values of *R_0_* in the OtherSE group did not differ from the noSE reference group (OR 1.42 [0.56-3.64], p=.460). No other RL model parameters emerged as significant predictors of ASA or OtherSE (all p>.250, except *β* which approached significance as a predictor of ASA, OR 0.09 [0.01-1.30], p=.078).

To determine whether *R_0_* could help predict upcoming ASA, above and beyond the contributions of other standard risk variables, **Table 4** summarizes the results of a GEE using *R_0_* as a predictor, adjusting for lifetime ASAs and contemporaneous SSI. Odds of an ASA increased as number of prior ASAs increased and as *R_0_* decreased.

**Table 4:** Results of GEE model, predicting actual suicide attempt (ASA) or other suicidal event excluding ASA (OtherSE) within 90 days based on estimated parameters from reinforcement learning (RL) model.

	Actual Suicide Attempt (ASA)			Other Suicidal Event (excluding ASA)		
	OR	95% CI	p-value	OR	95% CI	p-value
RL model: *R_0_*	**0.24**	**0.09-0.62**	**.003**	1.35	0.53-3.46	.527
SSI at time of RPLT testing	1.12	0.99-1.26	.070	0.98	0.91-1.06	.667
# Prior ASA (lifetime)	**1.58**	**1.07-2.34**	**.021**	0.69	0.45-1.05	.084

GEE includes session as repeated-measure. R_0_ is subjective value of ambiguous/neutral feedback. Bold indicates predictors for which p<.05.

## 4 Discussion

Cognitive function in general, and decision-making in particular, have long been recognized as part of the clinical profile of individuals who have attempted suicide (53). An important feature of decision-making is how we learn to adapt our responses based on feedback, such as rewarding and punishing outcomes. However, important questions remain regarding whether feedback-based learning is altered in suicidality, and if so whether it may prospectively predict whom among a group of at-risk individuals are likely to attempt suicide within an upcoming, near-term time window. The current study used a task that interleaved reward-based and punishment-based trials, and showed that better learning to obtain reward was associated with increased risk for suicide attempt within the next 90 days, and remained predictive even after controlling for other clinical variables (e.g., current suicidal ideation severity and history of prior attempts).

At first glance, it might appear paradoxical to observe *better* reward-learning performance in the ASA group. However, enhanced performance on reward-based trials of the RPLT could reflect a number of possible underlying cognitive mechanisms. Computational modeling, using the RL model, was used to examine latent cognitive parameters and revealed a key finding related to the no-feedback outcome (*R_0_*): specifically, upcoming ASA was associated with negative valuation of *R_0_*, and this provides an interpretation of the facilitated reward-based learning on the RPLT. We discuss these points next.

### 4.1 Subjective valuation of ambiguous outcomes, in those with upcoming ASA

Because the RPLT interleaves reward-based and punishment-based trials, *R_0_* is ambiguous: on a reward-based trial, *R_0_* represents failure to obtain reward, but on a punishment-based trial, *R_0_* represents successful avoidance of punishment. In fact, if the reward-based and punishment-based trials are trained in separate blocks, *R_0_* tends to be negatively-valued in the reward-based trials and positively-valued in the punishment-based trials (37). When both trial types are intermixed, different individuals can vary in their subjective valuation of *R_0_*, but among non-psychiatric populations, *R_0_* is typically valued as equivalent to a mild reinforcer, equivalent to a “glass half full” interpretation (*“At least I wasn’t punished!”*) (37, 54). In the current study, the noSE and OtherSE outcomes were similarly associated with a mildly positive valuation of the no-feedback outcome (*R_0_* >0). In contrast, upcoming ASA was associated with a negative valuation of the no-feedback outcome (*R_0_* <0), comparable to a mild punisher: a “glass half empty” interpretation (“*I failed to get rewarded*.”).

Importantly, this provides a possible mechanism that could underlie the facilitated reward-based learning observed on the RPLT in the ASA group. Specifically, valuation of *R_0_* <0 (i.e., negative valuation of ambiguous outcome) would tend to facilitate reward learning by increasing the contrast between outcomes of explicit reward *vs*. non-reward, compared to groups where *R_0_* is valued similarly to explicit reward. By the same token, *R_0_* <0 could conceivably impair punishment learning, decreasing the contrast between explicit punishment and non-punishment, and in fact **Figure 2B** suggests a slight (non-significant) decrease in optimal responding on punishment trials in the ASA group, compared to the noSE and OtherSE groups. Therefore, the association of ASA with better learning from reward trials on the RPLT could reflect an underlying bias towards negative valuation of neutral outcomes. One possibility is that *R_0_* therefore functions as a mediator of the relationship between reward learning and ASA. In the current dataset, *R_0_* was itself estimated based on the reward- and punishment-based learning during RPLT, but future studies could examine *R_0_* (derived from RPLT) as a possible mediator of reward learning on other tasks.

Beyond the RPLT task itself, these findings on negative evaluation of ambiguous feedback have implications for adaptation processes in daily life experienced by individuals at near-term risk of suicide. There may be a pointed deficit in learning from situational contingencies that do not provide explicit feedback. For example, one may respond adaptively to a stressor, yet not encounter a clear signal of “reward,” which may lead one to abandon that adaptive response. In general, given that the majority of daily life consists of events that are more ambiguous than an explicit reward or explicit punishment, a tendency to view all such ambiguous events as mildly punishing could promote feelings of helplessness and hopelessness – which in turn increase risk for suicidality. These results and interpretation broadly parallel the well-established finding of negative bias in depression (55) and of biases in social cognition in borderline personality disorder (56), which is a disorder characterized by high rates of suicidal behavior.

### 4.2 Relationship to prior research on feedback-based learning

We are aware of three other studies that applied an RL model to feedback-based learning in suicidality. The first two studies, by Dombrovski and colleagues (11, 17), used a simple forced-choice probabilistic task in which participants receive positive (rewarding) feedback following correct choices and negative (punishing) feedback following incorrect choices; those studies reported no differences between individuals with a history of prior suicide attempt compared to control groups on initial learning, in terms of either behavior or RL model variables, although the attempters were impaired at flexibly updating their response rules when contingencies were reversed. A third study, by Dixon-Gordon and colleagues (20) considered a probabilistic learning task in patients at high- or low-risk for suicide, and found no group differences on either learning accuracy or RL model variables, although group differences did emerge on the task after negative emotion induction. It remains an interesting open question whether similar results might emerge using the RPLT after a reversal and/or after negative emotion induction. However, the lack of group differences on basic response acquisition in these prior studies likely reflects both the simpler task designs and the fact that there were no ambiguous or neutral outcomes. It appears that the RPLT task design, with its frequent ambiguous feedback, allowed negative subjective interpretations of this ambiguous feedback to drive better reward-based learning in the ASA group.

In contrast to the current finding in patients at risk for suicide, several studies with the RPLT and other feedback-based learning tasks have found that depressive disorders tend to be associated with decreased learning from reward (25, 26) and/or increased learning from punishment (57). While depression and suicidality often co-occur and likely interact, the pattern of impaired learning from reward observed in depressed patients is therefore *opposite* from the enhanced reward learning found in the ASA group in the current study; further, as shown in the **Appendix** (**Supplementary Figure 10**), depression was associated with increased *LR+* in the current sample. It is important to note that, while depression is typically associated with decreased reward processing, it has also been shown that antidepressant medication moderates RPLT behavior (25), and it is likely that many of the patients in the current sample were on antidepressant medications, complicating interpretation. Despite this, MDD status – while positively associated with both ASA and OtherSE outcomes (see **Table 1**) – did not significantly improve prediction of these outcomes compared to RPLT variables alone (see **Appendix**: **Supplementary Table 6**).

Another prior study found a pattern of improved reward learning and reduced *R_0_* in Veterans with severe symptoms of PTSD compared to peers with few/no PTSD symptoms (21); this pattern is qualitatively similar to, although less extreme than, the results associated with ASA outcomes in the current study. However, in the current study, PTSD rates were actually somewhat lower in the ASA than OtherSE and noSE groups (**Appendix**: **Supplementary Figure 11**), and PTSD itself did not emerge as a predictor of either ASA or OtherSE (**Appendix**: **Supplementary Table 7**). Unfortunately, the earlier PTSD study did not collect information regarding suicidal behavior; nevertheless, it appears that PTSD alone does not adequately explain the results of the current study. Rather, it appears that upcoming ASA is associated with a unique profile of negative valuation of ambiguous outcomes. An important question for further research is whether this valuation is relatively fixed for one individual or may wax and wane over time (i.e., in the days or weeks leading up to an ASA *vs*. after the attempt is made). Additionally, altered reward learning may be a specific mechanism contributing to the increased risk for suicide attempt in those with PTSD, and should be explored in future studies.

Finally, in understanding the contrast between current findings and previous studies, it should also be emphasized that the earlier studies were retrospective, for example, comparing individuals with a history of prior suicide attempt against participants with no history of suicidal thoughts or behaviors (11, 17). In contrast, the current study prospectively followed participants already considered at high risk for suicide and who often had a history of one or more prior suicide attempts. It is therefore possible that reward-based learning among the ASA group in the current sample would appear impaired if contrasted to performance in a group of healthy volunteers with no prior history of suicidal thoughts or behaviors.

### 4.3 Limitations

There are several important limitations of this study. The first is that it is based on a small sample of Veterans, all of whom were characterized as high-risk; in fact, prior-year suicide-related hospital admission was an entry criterion for the study. Thus, it should not be assumed that the results obtained here would generalize to a broader population.

More importantly, even in this high-risk sample, there were a small number of ASAs and OtherSEs within the one-year follow-up period. The low base rate for suicide events, even in a high-risk sample, has been a key challenge for both clinical and basic research studies of suicide, and speaks to the need to replicate findings in additional samples.

Additionally, two ASAs occurred in our sample that could not be included in our prospective analysis, in one case because the ASA occurred more than 90 days after an RPLT session; in the other case, one individual had two separate ASAs within the 90-day follow-up window after an RPLT testing session, and so only the first one was counted. The current study was explicitly designed to have short 90-day follow-up periods, given that risk prediction will be most useful clinically with a narrow prediction window. In the interest of increasing the number of ASA outcomes, some prior studies have considered a longer follow-up window (e.g., 180 days (51, 52)), which of course allows more ASAs to be captured; however, even in those prior studies, the rate of ASA hovers around 10-15% over six months.

Although it remains true that the key findings in this study are driven by a small number of ASAs which occurred in a low number of participants, an important feature of the current study was repeated measurements across time, which allowed at least some initial examination of whether task performance and parameters are likely to change within-subjects, and thus to predict the timing of ASA. While extremely preliminary (based on only 3 patients), supplemental analysis (**Appendix**: **Supplementary Figure 9**) suggests that the same individuals show patterns indicative of upcoming ASA when tested on the RPLT shortly before ASA, but perform more like the noSE group when no ASA will occur in the next few months. If this can be validated in a larger sample, this would provide important information that could greatly aid targeting of clinical resources not just to identify individuals who are generally at high risk for suicide, but rather allowing these resources to be targeted in precise time windows of particularly high risk.

Given all of the above, the current results must be replicated in a larger dataset, and ideally in a sample large enough to validate in the predictive relationship in a hold-out group, as well as with frequent within-subject measurements to confirm whether facilitated reward learning (and negatively valued *R_0_*) may be state indicators that indicate periods of upcoming risk, rather than trait characteristics in a high-risk subgroup.

There are also limitations related to the sample itself. The focus on a Veteran sample meant that females were underrepresented, which limited power to detect possible sex differences. Prior studies with larger and more balanced samples have not to our knowledge reported sex effects on RPLT or related tasks (37, 54); however, sex differences in decision-making could well occur within the context of patients at risk for suicide. Emerging research also suggests a link between traumatic brain injury (TBI) and suicidality; however, we could not examine this in our current sample due to the absence of TBI cases in the ASA outcome group, likely due to small sample size (however, see **Appendix**: **Supplementary Figure 13**).

The current study also experienced challenges with follow-up assessments, including the interruption of in-person research testing during the COVID-19 pandemic; we were able to obtain follow-up outcome information on most participants enrolled before the shut-down, and only a few participants were censored due to death or withdrawal; of the withdrawn participants, one was by the participant’s request and the others were withdrawn by the research team due to participant incarceration or justice involvement, which is another common challenge in this population. There were also two participants whose RPLT data were excluded as non-compliant. There were no obvious differences in the censored or noncompliant subjects, compared to the rest of the sample, but differences might be detected in a larger sample. It is also important to recognize that no individuals died by suicide within the follow-up period; while most participants (78%) had a history of prior suicide attempt, the cognitive profile of those who die by suicide may be markedly different from those with severe suicidal ideation and/or suicidal behaviors who do not ultimately die by suicide.

Additionally, in the current sample there was insufficient information available regarding psychotropic medication use. Antidepressant medications have previously been shown to affect performance on the RPLT in patients with depressive disorder (25), specifically by reducing performance on punishment-based learning. Given the high rates of depression in the current sample, it remains unclear whether antidepressant use may have influenced the results, and if so whether this interaction differs in those with upcoming ASA or OtherSE. Performance on the RPLT and related tasks is also affected in patients with Parkinson’s disease on *vs*. off dopaminergic medication (24, 58) and in patients with current opiate addiction (22), further confirming the ability of psychoactive substances to affect how individuals process and respond to rewarding and punishing feedback. Important questions remain regarding whether similar effects of serotonergic, dopaminergic, opioid, and other medications would be evidenced in patients at risk for suicide. We do note, however, that in this sample, there did not appear to be differences related to lifetime abuse of opiates (see **Appendix**: **Supplementary Figure 12**).

## 5 Conclusion

Despite these limitations, the results suggest an intriguing feature of cognition that appears to be associated with, and predictive of, upcoming near-term ASA in individuals considered at risk for suicide. Given that much of everyday life is neither explicitly rewarding nor explicitly punishing, the vast majority of human experience may be comparable to ambiguous or neutral reinforcement; if these events are viewed negatively (*R_0_* <0), this could have real-world implications, particularly in understanding individuals’ negative expectations and interpretations of neutral situations. In fact, a focus on reducing negative bias has been the hallmark of evidence-based interventions such as Cognitive Behavioral Therapy for suicidality and depression. Feedback-based learning tasks, with concomitant modeling to extract latent parameters, may therefore also assist in objective assessment of these cognitive biases, and their potential modification by therapy, in those at risk for suicide.

## Data Availability

The datasets presented in this study can be found in online repositories. The names of the repository/repositories and accession number(s) can be found below: A deidentified dataset supporting this work, along with the behavioral task software, modeling code, and scripts for statistical analysis, are posted on Open Science Framework (OSF) at https://osf.io/p328a/.
