# Supplementary material for: Negative valuation of ambiguous feedback may predict near-term risk for suicide attempt in Veterans at high risk for suicide
Source: Front Psychiatry. 2025 Jan 30;15:1492332. doi: 10.3389/fpsyt.2024.1492332 (PMC11821650; doi:10.3389/fpsyt.2024.1492332)
Supplement: Supplementary file 1 [file DataSheet1.docx]

# Appendix

# Negative valuation of ambiguous feedback may predict near-term risk for suicide attempt in Veterans at high risk for suicide:

# Detailed Methods and Supplementary Results

Catherine E. Myers, Rokas Perskaudas, Vibha Reddy, Chintan V. Dave, John G. Keilp,

Arlene King, Kailyn Rodriguez, Lauren St. Hill, Rachael Miller & Alejandro Interian

# Contents:

## 6.1. Detailed Methods: Recruitment, Screening and Data Collection

## 6.2. Detailed Methods: Computational Model

## 6.3. Supplemental Results

## 6.4. Supplemental References

## 6.1. Detailed Methods: Recruitment, Screening and Data Collection

#### 6.1.1. Recruitment

To identify potential participants, study staff conducted regular screening of acute psychiatry admissions records at VA New Jersey Health Care System (VANJHCS) for patients with admission related to suicidal ideation or behavior, supplemented by review of Veterans Health Administration (VHA) electronic medical records to identify Veterans with a prior year admission to an acute psychiatry ward or emergency department following either a suicide attempt (SA) or severe suicidal ideation (SI), and who appeared to meet other eligibility criteria.

For potential participants identified during an in-patient stay on the VANJHCS acute psychiatric ward or a residential stay at VANJHCS, a study clinician (Masters- or Doctoral-level study personnel with formal mental health training) approached the individual either in person or by telephone, to briefly describe the study and ask whether the individual might be willing to participate. Potential participants not currently on the VANJHCS campus were contacted via IRB-approved recruitment letter, which briefly described the study and provided information about how to opt-out of further contact; if opt-out was not triggered, study staff contacted potential participants by telephone at least one week later to explain the study and answer any questions.

If the potential participant agreed, screening was conducted and, if inclusion/exclusion criteria were satisfied, the participant was enrolled and baseline testing either occurred or was scheduled for a convenient time.

#### 6.1.2. Screening

Participants were screened for lifetime history of suicide attempt and suicidal ideation severity using the Columbia Suicide Severity Rating Scale (C-SSRS) (1) and for current (past-week) suicidal ideation severity using the Beck Scale for Suicidal Ideation (SSI) (2). For inclusion, participants had at least one of (a) past-year actual, interrupted or aborted suicide attempt, (b) past-year preparatory behavior, or (c) severe SI defined as 4+ on the SSI.

Exclusion criteria were severe hallucinations or delusions, disorganized or disruptive behavior, medical instability (e.g. acute drug withdrawal), or lack of cognitive capacity to complete the study tasks and provide informed consent. Screening related to medical/clinical exclusions was based on the Mini International Neuropsychiatric Interview (MINI) (3), a brief structured interview to assess diagnostic criteria for DSM-IV Axis 1 and ICD-10 psychiatric diagnoses. Note that current or past medical or psychiatric diagnoses would not automatically trigger exclusion. For participants screened in person, cognitive competency was assessed via the Montreal Cognitive Assessment (MoCA) (4); for participants screened by telephone, the MoCA-Blind v. 8.1 was used, which eliminates visual elements. Following prior studies with Veterans (5), we used a cut-off score of 20+ on the 30-point MoCA (or 15+ on the 22-point MoCA-Blind); individuals scoring below this level were considered to have cognitive impairment and were excluded from participation.

Participants were also required to be over age 18, to have English fluency sufficient to provide informed consent and complete tasks and questionnaires delivered in English, and to have sufficient sensorimotor function to view the computer screen and press keyboard keys (using any normal corrective technology, e.g., glasses/contact lenses).

Participants meeting the screening criteria were enrolled in the study, and written informed consent was obtained. Information obtained during screening (C-SSRS, SSI, MINI, MoCA or MoCA-Blind, and medical record information) then became part of the study data for that participant. Participants were reimbursed for participation at the rate of $50 for baseline testing (Session 1) rising to $70, $80, $90, and $100 for Sessions 2-5. Participants (excluding in-patients and those in VANJHCS residential programs) were also reimbursed for travel to/from the testing site.

#### 6.1.3. Baseline Testing (Session 1)

Baseline testing (Session 1) lasted approximately 2 hours, and included clinical interview, self-report questionnaires, and neurocognitive testing. Where possible, the session was completed in a single sitting, but on occasion the interview and neurocognitive testing took place on subsequent days.

The clinical interview was administered by a study clinician and included updated Beck SSI and C-SSRS (if screening had occurred more than 1 day previously), as well as brief medical history, including the Brief Traumatic Brain Injury Screen (BTBIS) (6) modified to include injuries sustained outside of as well as during military deployment; the Brown-Goodwin Lifetime History of Aggression (LHA) Scale (7,8); current (prior 30 days) alcohol, tobacco, and substance use; and military history including the Combat Exposure Scale (CES) (9). The interviewer also queried social relationships and family history (including rent stress/homelessness, living alone vs. with others, etc.).

The self-report questionnaires were administered via paper-and-pencil, and included a demographic questionnaire (age, gender, race/ethnicity, education, employment and marital status); the Beck Depression Inventory (BDI-II) (10) to asses severity of current depression symptoms; the Beck Hopelessness Scale (BHS) (11) to assess hopelessness and negative attitudes about the future; and additional questionnaires not reported here (including questionnaires related to life stressors and negative thoughts).

Participants also completed the reward- and punishment-learning task (RPLT), described further below, and one or more additional neurocognitive tasks not reported here (including a Go/No-Go test of motor inhibition, a recognition memory test of short-term recall, an emotional Stroop test to assess distractibility, and an implicit association test to assess association of self with concepts of death and suicide).

#### 6.1.4. Follow-up Testing (Sessions 2-5)

Follow-up testing sessions lasting approximately 1.5-2 hours each were scheduled at approximately 3-month intervals over one year (i.e., approximately 3, 6, 9, 12 months after Session 1). These included updated C-SSRS, current SSI (covering past week), and updated information on medical treatments, social relationships, and medication and drug use; self-report questionnaires (BDI-II, BHS) covering time since last research visit; and neurocognitive testing.

Telephone check-ins were scheduled at the halfway point between testing sessions, to maintain contact and check on participant welfare, including facilitating referral to mental health services if appropriate.

If a participant could not complete one or more follow-up sessions, attempts were made to complete the clinical interview (particularly C-SSRS and SSI) by telephone; in a few cases, self-report packets were sent and returned by mail. Particular effort was made to capture at least C-SSRS at Session 5, since this provided information on study outcomes during the prior year.

During the COVID-19 pandemic, a year-long administrative hold on face-to-face research encounters at VANJHCS (Spring 2020-Spring 2021) interrupted planned follow-up of the first cohort of 30 patients. Where possible, partial follow-up sessions were conducted by telephone and/or mail during this period (clinical interview and self-report questionnaires). Additionally, participants who had granted permission for re-contact at the time of initial informed consent were re-contacted after Spring 2021 and asked if they would be willing to re-enroll for another year of follow-up. Five participants agreed, and subsequently completed one or more additional testing sessions, which were included in the dataset along with outcomes occurring in the 90 days after those testing sessions.

## 6.2. Detailed Methods: Computational Model

#### 6.2.1. RL Model Implementation

The RL model was a *Q*-learning model adapted from methods in (12,13), programmed in the C programming language. The *Q*-learning model (14,15), schematized in **Figure 4**, assumes that the model learns a *Q*-value or weight associated with each possible response to a stimulus (category *A* or *B*). When a new stimulus *S* is presented, the model evaluates each possible response *r* to that stimulus as:

*E_r,S_ = exp(Q_r,S_/β)* (Eq. 1)

Here, *β* is a “temperature” parameter: low values increase the tendency to exploit (choose response with highest *Q*-value), while large values increase the tendency to explore other possible responses. The model then selects a response using a softmax logistic function:

*Prob(response=A) = E_A,S_/(E_A,S_+E_B,S_)* (Eq. 2)

*Prob(response=B) = 1-Prob(response=A)* (Eq. 3)

External feedback *R* is then provided (+1 for reward, -1 for punishment, or *R_0_* for the neutral/ambiguous outcome), and prediction error *PE* is computed as the difference between actual reinforcement *R* and the expected value *Q*. *Q*-values for the selected response *r* are then updated as:

*Q_r,S_ 🡨 Q _r,S_ + PE*LR* (Eq. 4)

where *LR* is a learning rate, set to *LR*+ for better-than-expected outcomes (*PE*>0) or *LR*- for worse-than-expected outcomes (*PE*<0); when *PE*=0, the outcome is predicted perfectly and there is no learning. Thus, model behavior is defined by the values of free parameters *LR*+, LR*-,* *β*, and *R_0_*.

| 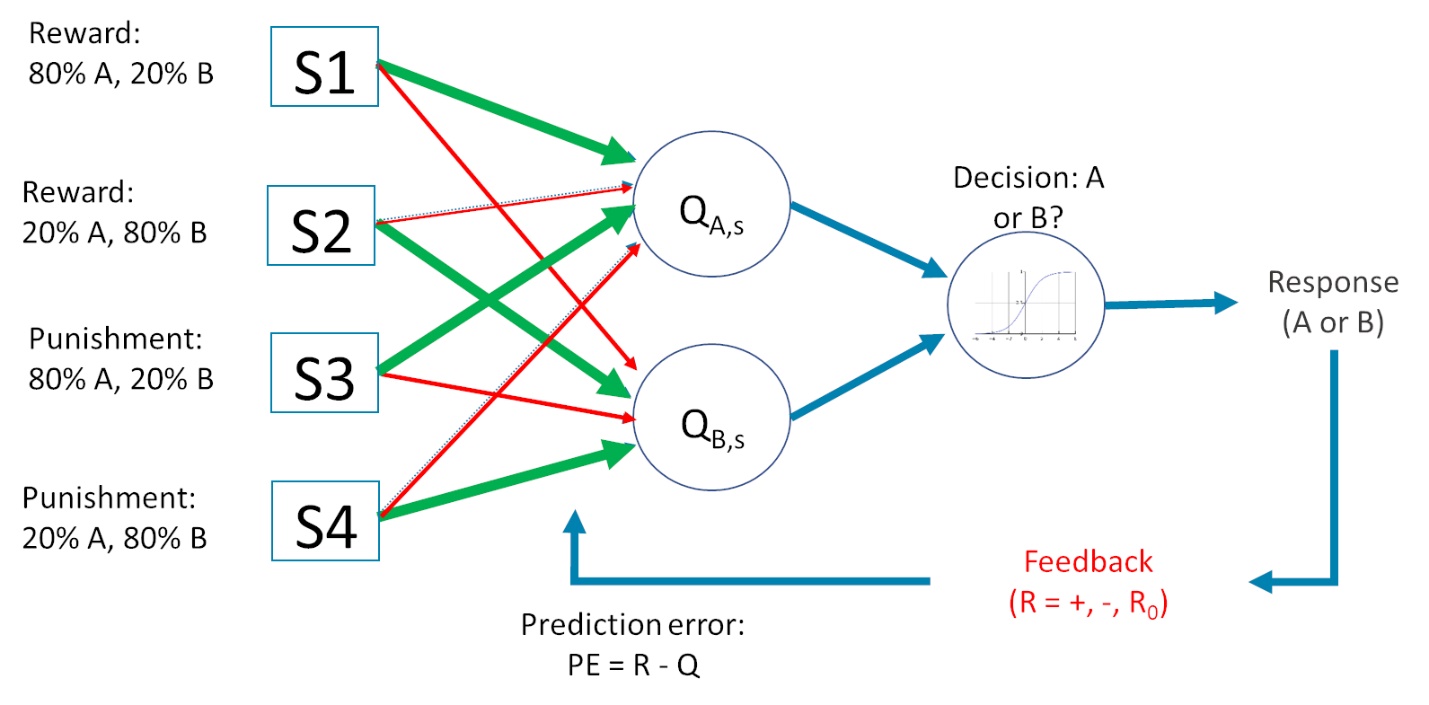 |
| --- |
| **Figure 4**. Schematic of Q-learning model; on each trial one stimulus (S1-S4) is presented; *Q*-values for that stimulus paired with each possible response (here, category A or B) are fed to a decision algorithm; a response is chosen and emitted, feedback (*R*) provided, and prediction error (*PE*) used to update the *Q*-values for the selected stimulus-response pair. |

To simulate individual-level participant data, the model is presented with the same stimuli in the same order as the participant; and *Prob(response=A)* is calculated. The model is then forced to execute the same response as the participant did at that trial, and receive the same feedback, on the basis of which it adjusts *Q-*values for the current stimulus-response pairing. Grid search is used to optimize parameter values to minimize negative log-likelihood estimate (*negLLE*), i.e., maximizing the probability that the model’s response on each trial would match that of the participant. Learning rates *LR*+ and *LR*- and temperature *β* are allowed to range 0..1, and subjective value of neutral feedback *R_0_* is allowed to range from -1 (same as explicit punishment) to +1 (same as explicit reinforcement). The specific configuration of parameter values that, together, minimize *negLLE* are reported as parameter estimates for that participant.

The RL model software was custom-written in C and called via a wrapper written in R v. 4.3.1, using the *Rcpp* package (16). Each datafile takes ~15 sec to simulate using R 4.1.0 on a Dell desktop PC (Intel Core i5-10500 3.10 GHz with 16GB RAM) under Windows 10 Enterprise (single core). The model software is available at: <https://osf.io/h7q8m/?view_only=03ddac2e39d64e8382e46c3e34e5ef28>.

#### 6.2.2. Model Comparison Studies

In addition to the model with four free parameters *LR+, LR-, β* and *R_0_*, we also considered a more complex model that implemented a working memory trace *wm* that stored the most recent response made to each stimulus. There were therefore six parameters in this expanded model: *LR+, LR-, β, R_0_*, as well as *P*, which ranges -1..+1 and describes tendency either to perseverate by repeating the last response, regardless of outcome (*P* 🡪 +1), or display spontaneous alternation (*P* 🡪 -1); and a decay parameter *d* that ranges 0..1. The expectancy for each possible response *r* to the current stimulus *S* was recalculated to include this working memory trace:

*E_r,S_=exp(Q_r,S_/β + P*wm[r,S])* (Eq. 5)

Here, *wm[r,S]* was set to 1 if the current trial included stimulus *S* and response *r*, and otherwise decayed across trials as *wm[r,S]🡨wm[r,S]*d*.

Omnibus model comparisons between the two models, using the Akaike Information Criterion (AIC) (17), which compares model goodness-of-fit, indicated that the simpler 4-parameter model (mean AIC=100.8) provided a relatively accurate and more parsimonious description of the data than the 6-parameter model (mean AIC=101.7), while the relative likelihoods were essentially equal (4-parameter model: 0.501; 6-parameter model: 0.499). Accordingly, the 4-parameter model was retained and is reported.

Nevertheless, for comparison, **Figure 5** shows that the 6-parameter model also captures the “sign flip” in estimates of *R_0_*, such that the noSE and OtherSE groups tend to value the ambiguous/neutral outcome as mildly reinforcing, while the ASA group values it as mildly punishing (compare Figure 3). However, GEE models including RL6 parameter estimates as potential predictors of 90-day outcomes revealed no significant predictors of either ASA or OtherSE (all p>.100; results not shown).

| 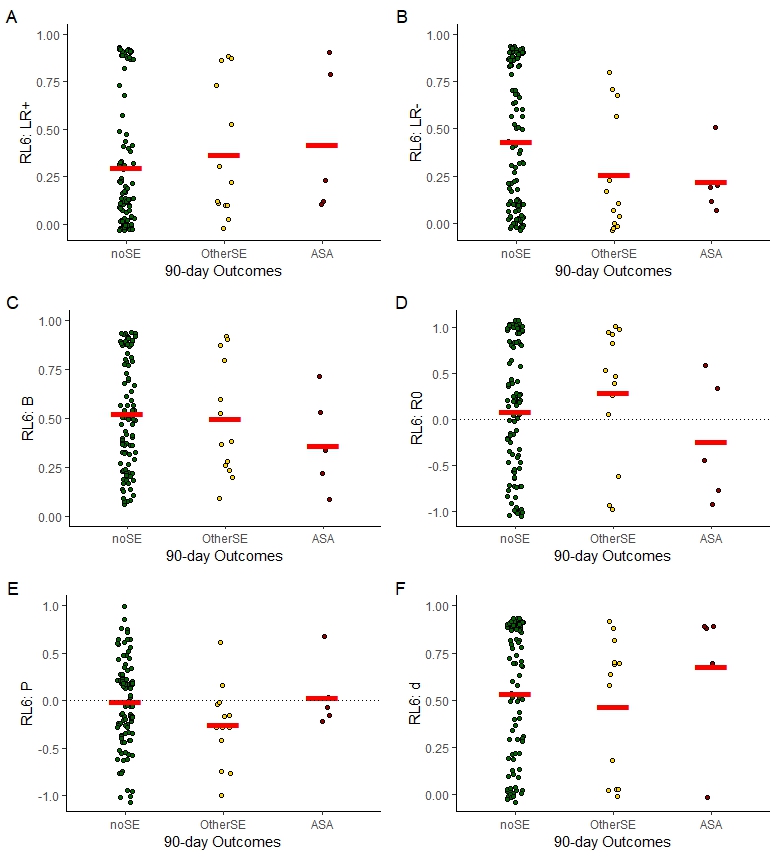 |
| --- |
| **Figure 5**. Mean parameter estimates in the expanded 6-parameter RL model (“RL6”), by 90-day outcome group, for comparison with estimates from the simpler 4-parameter model shown in Figure 3. *P, d*=perseveration and decay parameters in the model; for this and subsequent figures, other abbreviations/conventions as in Figures 2 & 3 of main text. |

#### 6.2.3. Predictive Check and Parameter Recovery Studies

After determining parameter estimates for each RPLT data file using the RL4 model, predictive check and parameter recovery studies were performed. First, using the estimated parameters *LR+, LR-, β,* and *R_0_* for each datafile (“generating” parameters), an RL model was constructed and trained on the RPLT task, using the same 80 trials as the behavioral task. At each trial, the model generated a response and updated Q-values, using Eq. 1-4 above. The trial-by-trial responses were saved in “simulated” data files, which could then be analyzed for percent optimal responding, in the same way as the actual data files. **Figure 6** shows group-level correspondence between the actual data and simulated data: specifically, higher rates of optimal responding to reward-based, but not punishment-based, stimuli in the ASA group than in the OtherSE and noSE groups.

| 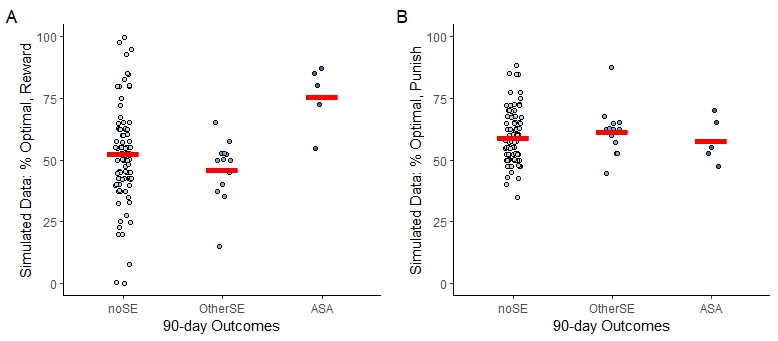 |
| --- |
| **Figure 6**. Results from predictive check: Estimated RL parameter values from each RPLT data file (“generating” parameters) were used to construct an RL model that was then tested on the RPLT task, to generate “simulated” data files that can be compared to the actual behavioral data. The simulated data accurately captures higher rate of optimal responding to reward-, but not punishment-based, stimuli in the ASA group, compared to the other groups (compare Figure 2 of main text). |

Next, the parameter estimation procedures were applied to the simulated datafiles, and the “recovered” parameter estimates *LR+, LR-, β,* and *R_0_* recorded for each. **Figure 7** shows that the “recovered” parameters show group-level correspondence with the “generating” parameters, particularly the pattern of *R_0_<0* (valuing the neutral ambiguous outcome as similar to a mild punisher) in the ASA but not OtherSE or noSE groups (compare Figure 3 of main text).

| 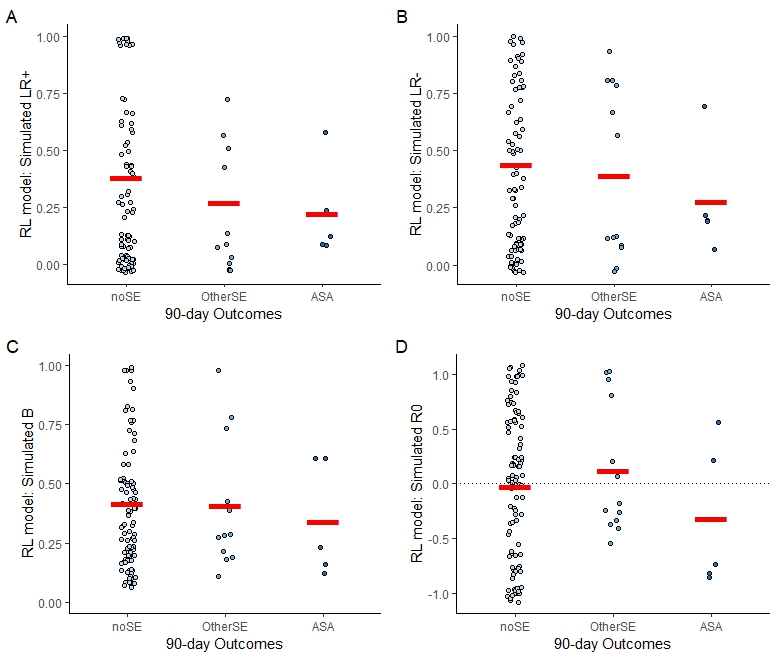 |
| --- |
| **Figure 7**. Results from parameter recovery: The parameter estimation procedures were applied to each “simulated” data file, to see if the generating parameters could be accurately recovered. Results show that the recovered parameters correctly capture features of the generating parameters, including negatively-valued *R_0_* in the ASA group (compare Figure 3D). |

## 6.3. Supplemental Results

#### 6.3.1. Supplementary analyses: Post-hoc analyses of individual differences and within-subject effects

Following the observation (Figure 2 and Table 3) that ASA could be predicted based on RPLT (optimal responding on reward-based trials) and ASA history, important questions arise regarding whether there are clinical and demographic variables characterizing high-risk individuals, and whether the observed behavior represent state changes in the run-up to an event or trait variables that characterize high-risk individuals across time. While our limited dataset does not allow full examination of these questions, we conducted two supplemental analyses to attempt to characterize (a) individual differences among patients who would vs. would not experience an upcoming ASA, and (b) within-subject differences in participants when there was vs. was not imminent ASA. These results should be considered extremely preliminary due to small sample size, but are presented as suggestive of both between-subjects effects (higher-risk individuals) and within-subjects effects (state changes in RPLT performance).

To address individual differences, we constructed a subset including only session 1 data (one observation per patient): this included 3 individuals with an ASA in the next 90 days, 6 with an OtherSE, and 45 with noSE in the next 90 days, and we confirmed visually that the observed pattern of better reward-based (but not punishment-based) learning and more prior ASAs in the patients with upcoming ASA, compared to those with upcoming OtherSE or noSE, is conserved in this reduced sample (**Figure 8**). This suggests that the effects observed in the GEE (Table 4), which embeds nesting by subjects, is not merely an artifact of the repeated-measures design, in which some patients contribute multiple data points to the dataset.

| 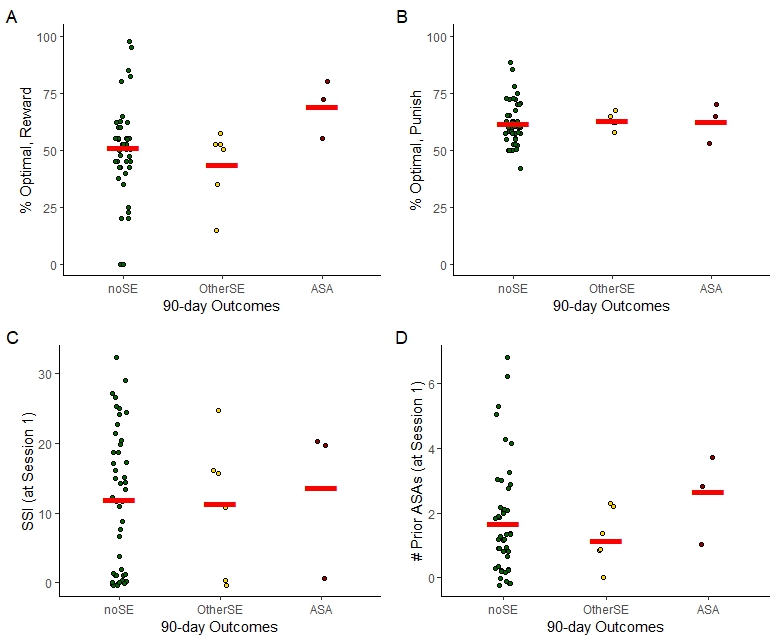 |
| --- |
| **Figure 8**. Percent optimal responding to (A) reward-based stimuli and (B) punishment-based stimuli on the RPLT, as well as (C) SSI and (D) number of prior ASAs, all assessed at session 1 only (n=54, one datapoint per subject), as a function of outcome within 90 days post session 1. Although preliminary due to extremely small sample size – only three individuals had an ASA within 90 days post session 1, and an additional 6 had an OtherSE (excluding ASA) within 90 days post session 1 -- the same basic pattern obtains as in the larger dataset of Figure 2 (which includes repeated-measures): namely, greater optimal responding to reward trials and higher lifetime ASAs in those subjects with upcoming ASA. Abbreviations/conventions as in Figure 2. |

Turning to within-subject effects, a key question is whether the individuals who experience an ASA outcome have generally higher reward-based learning across sessions (i.e., a relatively stable or “trait” risk factor), or if RPLT taps into a “state” variable that can help predict the timing of upcoming ASA within an individual. In consideration of this question, we considered all available RPLT data from the patients who experienced at least one ASA within a 90-day window after RPLT testing: i.e., three patients who together completed 8 RPLT testing sessions, 5 followed by ASA and 3 followed by noSE (none were followed by OtherSE). Despite the very small sample size, it appears that there is greater reward learning by those patients in RPLT testing sessions that precede an ASA, compared to RPLT testing sessions that precede noSE (**Figure 9**); further, the average performance on reward (and punishment) learning preceding noSE is comparable to that observed in the larger sample of noSE (compare Figure 2).

For comparison, we also considered all available RPLT data from the 11 patients who experienced at least one OtherSE (but no ASAs) in the 90-day window after RPLT testing: i.e., 11 patients who together completed 30 RPLT sessions: 13 followed by OtherSE and 17 followed by noSE (none were followed by ASA). Here, there appears to be no evidence of differential performance on reward (or punishment) learning in RPLT sessions that precede an Other SE, compared to those that precede noSE.

In drawing conclusions from Figures 8 and 9, it is paramount to stress the exploratory nature of the analysis and the extremely small sample sizes. Nevertheless, Figure 9 in particular suggests that the increased reward learning associated with ASA in the larger sample (Figure 2 and Table 3) is not merely a “trait” effect by which some individuals are both better at reward-based learning and at higher overall risk of ASA. Rather, it suggests that there may be variability in RPLT performance across time, with improved reward-based learning occurring in a time window leading up to an ASA, compared to time windows where no ASA is imminent.

| 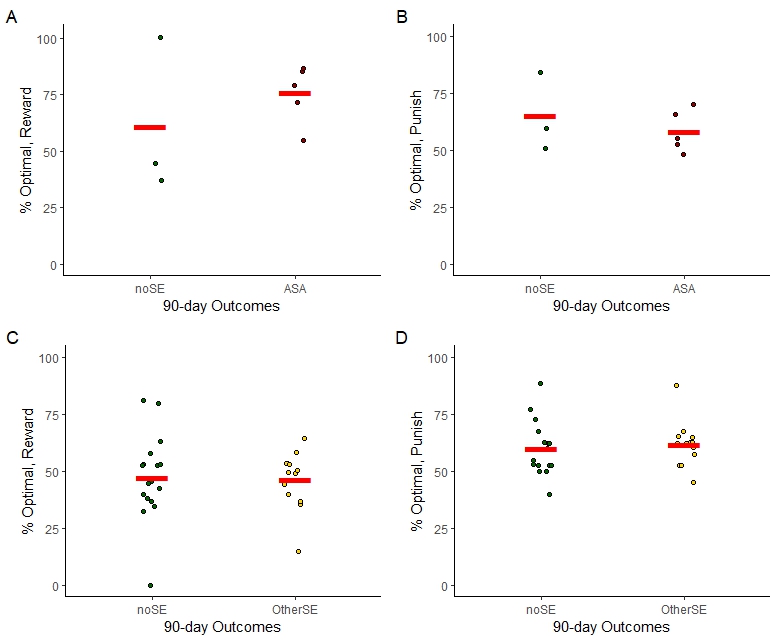 |
| --- |
| **Figure 9**. (A,B) RPLT behavioral variables from all available sessions in just those three participants who experienced at least one ASA within a 90-day window after RPLT testing. These three participants together completed 8 RPLT testing sessions: 5 followed by ASA and 3 followed by noSE (none were followed by OtherSE). There again appears to be higher level of reward learning in sessions preceding ASA than in sessions preceding noSE. For comparison, (C,D) shows RPLT behavioral variables in just those 11 participants who experienced at least one OtherSE within a 90-day window after RPLT testing. These 11 participants together completed 30 RPLT testing sessions: 13 followed by OtherSE and 17 by noSE (none were followed by ASA). Abbreviations/conventions as in Figure 2. |

#### 6.3.2. Supplementary analyses: OtherSE recency

All participants had 1+ prior year SE (ASA or OtherSE) as part of study entry criteria; however, in some cases, RPLT testing occurred in the acute aftermath of an SE (e.g., while participant was in-patient in acute psychiatry ward following a suicide-related admission). Accordingly, GEE models were run on the RPLT behavioral variables and RL model estimates, controlling for whether subjects had prior-week SE (vs. prior-year SE excluding prior-week SE). Results are shown in **Table 5**. In brief, percent optimal responding on reward-based trials was again associated with increased risk of ASA, as was decreased *R_0_*; prior-week SE did not emerge as a significant predictor of either ASA or OtherSE in this sample.

| ***Table 5****. Results of GEE model, predicting 90-day outcomes based on (a) RPLT behavioral variables or (b) RL model variables, and 1+ SE in week prior to RPLT testing (note all participants had 1+ SE in prior year, as study entry criterion).* |
| --- |
| \|  \| Actual Suicide Attempt  (ASA) \| \| \|  \| Other Suicidal Event  (excluding ASA) \| \| \| \| \| \| --- \| --- \| --- \| --- \| --- \| --- \| --- \| --- \| --- \| --- \| \|  \| OR \| 95% CI \| p-value \|  \| OR \| \| 95% CI \| p-value \| \| 1. *Predicting outcome based on RPLT and prior-week SE* \|  \|  \|  \|  \|  \| \|  \|  \| \| RPLT: % optimal, reward-based trials \| **1.08** \| **1.04-1.12** \| **<.001** \|  \| 0.98 \| \| 0.96-1.01 \| .142 \| \| RPLT: % optimal, punishment-based trials \| 0.95 \| 0.89-1.02 \| .135 \|  \| 1.02 \| \| 0.97-1.08 \| .399 \| \| Prior week SE (reference level=no) \| 0.47 \| 0.03-7.62 \| .596 \|  \| 0.80 \| \| 0.22-2.86 \| .729 \| \|  \|  \| \| \|  \|  \| \| \| \| \| \| 1. *Predicting outcome based on RL model and prior-week SE* \|  \|  \|  \|  \|  \|  \| \|  \| \| RL model: *R_0_* \| **0.48** \| **0.28-0.82** \| **.006** \|  \| 1.20 \| 0.49-2.95 \| \| .684 \| \| Prior week SE (reference level=no) \| 0.75 \| 0.07-8.61 \| .818 \|  \| 0.80 \| 0.22-2.92 \| \| .730 \| |

#### 6.3.3. Supplementary analyses: Depression

Prior work with the RPLT has shown that patients with depressive disorders (25,26,64) show impairment on reward-based but not punishment-based learning (18,19). Accordingly, we also investigated whether major depressive disorder (MDD) could account for the current results. In fact, patients with MDD showed no apparent impairment in reward-based learning on RPLT as well as increased *LR+* in the RL model, compared to those without MDD (**Figure 10**). However, MDD status did not significantly contribute to GEE models using either the behavioral variables or *R_0_* to predict 90-day outcomes (**Table** **6**), although percent optimal responding on reward-based trials was again associated with increased risk of ASA, as was decreased *R_0_*.

| ***Table 6****. Results of GEE model, predicting 90-day outcomes based on RPLT behavioral variables and major depressive disorder (MDD). Other abbreviations/conventions as in Tables 2 and 3.* |
| --- |
| \|  \| Actual Suicide Attempt  (ASA) \| \| \|  \| Other Suicidal Event  (excluding ASA) \| \| \| \| \| \| --- \| --- \| --- \| --- \| --- \| --- \| --- \| --- \| --- \| --- \| \|  \| OR \| 95% CI \| p-value \|  \| OR \| \| 95% CI \| p-value \| \| 1. *Predicting outcome based on RPLT and MDD* \|  \|  \|  \|  \|  \| \|  \|  \| \| RPLT: % optimal, reward-based trials \| **1.08** \| **1.03-1.12** \| **<.001** \|  \| 0.98 \| \| 0.96-1.00 \| .108 \| \| RPLT: % optimal, punishment-based trials \| 0.95 \| 0.89-1.01 \| .123 \|  \| 1.02 \| \| 0.97-1.08 \| .425 \| \| MDD \| 2.18 \| 0.18-25.81 \| .538 \|  \| 1.27 \| \| 0.40-4.00 \| .684 \| \|  \|  \|  \|  \|  \|  \| \|  \|  \| \| 1. *Predicting outcome based on RL model and MDD* \|  \|  \|  \|  \|  \| \|  \|  \| \| RL model: *R_0_* \| **0.46** \| **0.27-0.78** \| **.004** \|  \| 1.19 \| 0.49-2.88 \| \| .707 \| \| MDD \| 2.26 \| 0.18-27.77 \| .524 \|  \| 1.22 \| 0.37-4.06 \| \| .744 \| |

| 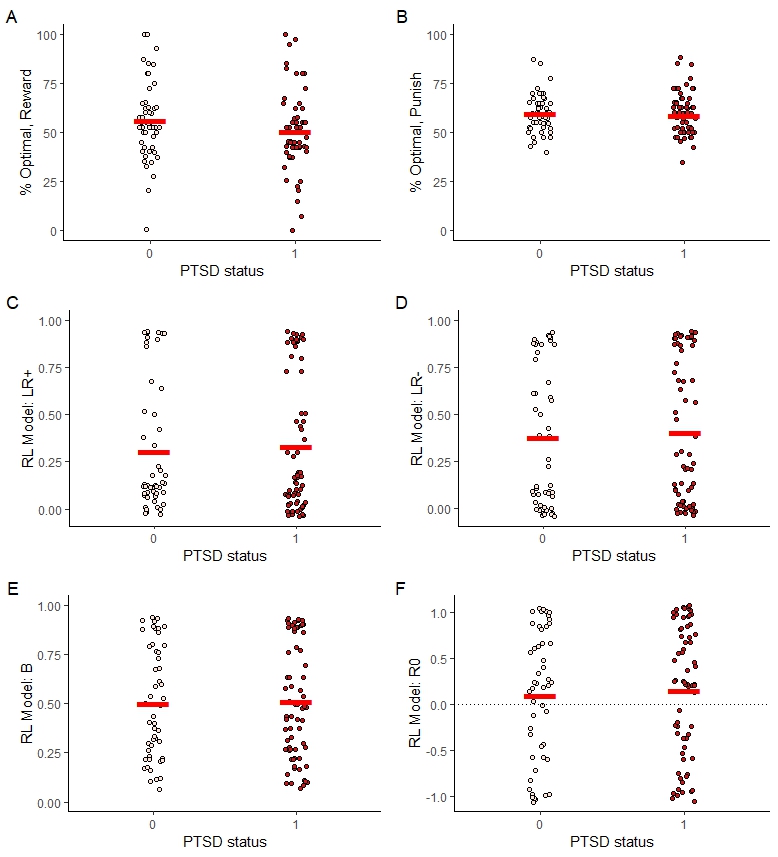 |
| --- |
| **Figure 10**. RPLT behavioral variables and RL model estimates, as a function of major depressive disorder (MDD) status (1=yes, 0=no). Other abbreviations/conventions as in Figures 2 & 3. |

#### 6.3.4. Supplementary analyses: PTSD

Prior work has indicated that Veterans with severe PTSD symptoms show enhanced reward-based learning on RPLT, along with reduced (although still positive) values of *R_0_*, compared to a group with few/no PTSD symptoms (13). Accordingly, we also investigated whether PTSD status could account for the current results. In the current study, however, participants with PTSD showed neither increased optimal responding to reward-based trials on RPLT, nor decreased *R_0_*, compared to those without PTSD (**Figure 11**), and PTSD status did not significantly contribute to GEE models using either the behavioral variables or *R_0_* to predict 90-day outcomes (**Table 7**), although percent optimal responding on reward-based trials was again associated with increased risk of ASA, as was decreased *R_0_*.

| ***Table 7****. Results of GEE model, predicting 90-day outcomes based on (a) RPLT behavioral variables or (b) RL model variables, and post-traumatic stress disorder (PTSD).* |
| --- |
| \|  \| Actual Suicide Attempt  (ASA) \| \| \|  \| Other Suicidal Event  (excluding ASA) \| \| \| \| \| \| --- \| --- \| --- \| --- \| --- \| --- \| --- \| --- \| --- \| --- \| \|  \| OR \| 95% CI \| p-value \|  \| OR \| \| 95% CI \| p-value \| \| 1. *Predicting outcome based on RPLT and PTSD* \|  \|  \|  \|  \|  \| \|  \|  \| \| RPLT: % optimal, reward-based trials \| **1.08** \| **1.03-1.12** \| **<.001** \|  \| 0.98 \| \| 0.96-1.00 \| .109 \| \| RPLT: % optimal, punishment-based trials \| 0.94 \| 0.89-1.00 \| .052 \|  \| 1.02 \| \| 0.97-1.08 \| .414 \| \| PTSD (reference level=no) \| 0.18 \| 0.02-2.04 \| .166 \|  \| 0.51 \| \| 0.15-1.72 \| .276 \| \| 1. *Predicting outcome based on RL model and PTSD* \|  \|  \|  \|  \|  \|  \| \|  \| \| RL model: *R_0_* \| **0.48** \| **0.28-0.82** \| **.007** \|  \| 1.21 \| 0.49-2.98 \| \| .685 \| \| PTSD (reference level=no) \| 0.16 \| 0.01-1.85 \| .143 \|  \| 0.54 \| 0.16-1.81 \| \| .322 \| |

| 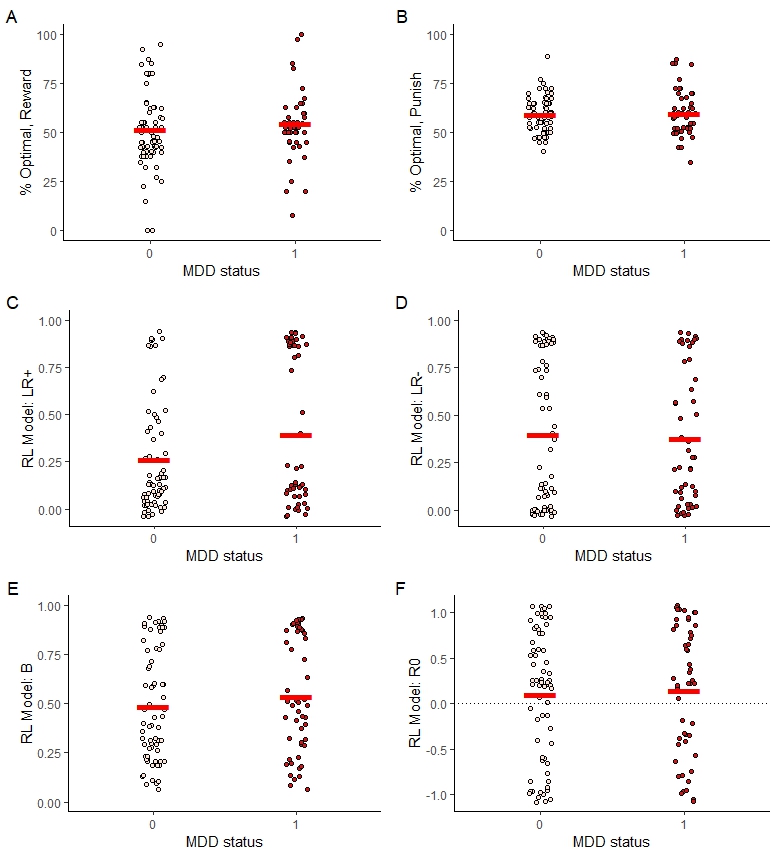 |
| --- |
| **Figure 11**. RPLT behavioral variables and RL model estimates, as a function of PTSD status (1=yes, 0=no). Other abbreviations/conventions as in Figures 2 & 3. |

#### 6.3.5. Supplementary analyses: Opioid Use

Prior work with the RPLT has also examined individuals with current addiction to opiates (20). Although only 3 participants in the current study reported past-month use of opiates to get high (none of whom had an upcoming ASA), 18 of 60 participants reported some lifetime history of opioid use (including any use of heroin, morphine, or prescription painkillers to get high). In fact, patients with history of opioid use showed no apparent difference from non-users on RPLT, although they did appear to have reduced LR+ in the RL model (**Figure 12**). History of opioid use did not significantly contribute to GEE models using either the behavioral variables or R_0_ to predict 90-day outcomes (**Table** **8**), although percent optimal responding on reward-based trials was again associated with increased risk of ASA, as was decreased R_0_.

| ***Table 8****. Results of GEE model, predicting 90-day outcomes based on RPLT behavioral variables and history of opioid use to get high.* |
| --- |
| \|  \| Actual Suicide Attempt  (ASA) \| \| \|  \| Other Suicidal Event  (excluding ASA) \| \| \| \| \| \| --- \| --- \| --- \| --- \| --- \| --- \| --- \| --- \| --- \| --- \| \|  \| OR \| 95% CI \| p-value \|  \| OR \| \| 95% CI \| p-value \| \| 1. *Predicting outcome based on RPLT and MDD* \|  \|  \|  \|  \|  \| \|  \|  \| \| RPLT: % optimal, reward-based trials \| **1.09** \| **0.89-1.00** \| **<.001** \|  \| 0.98 \| \| 0.96-1.00 \| .103 \| \| RPLT: % optimal, punishment-based trials \| **0.94** \| **0.89-1.00** \| **.043** \|  \| 1.02 \| \| 0.97-1.08 \| .399 \| \| Opioid use \| 2.48 \| 0.77-7.97 \| .126 \|  \| 1.33 \| \| 0.62-2.88 \| .467 \| \|  \|  \|  \|  \|  \|  \| \|  \|  \| \| 1. *Predicting outcome based on RL model and MDD* \|  \|  \|  \|  \|  \| \|  \|  \| \| RL model: *R_0_* \| **0.47** \| **0.29-0.77** \| **.003** \|  \| 1.21 \| 0.50-2.89 \| \| .676 \| \| Opioid use \| 1.74 \| 0.46-6.57 \| .411 \|  \| 1.26 \| 0.58-2.74 \| \| .558 \| |

| 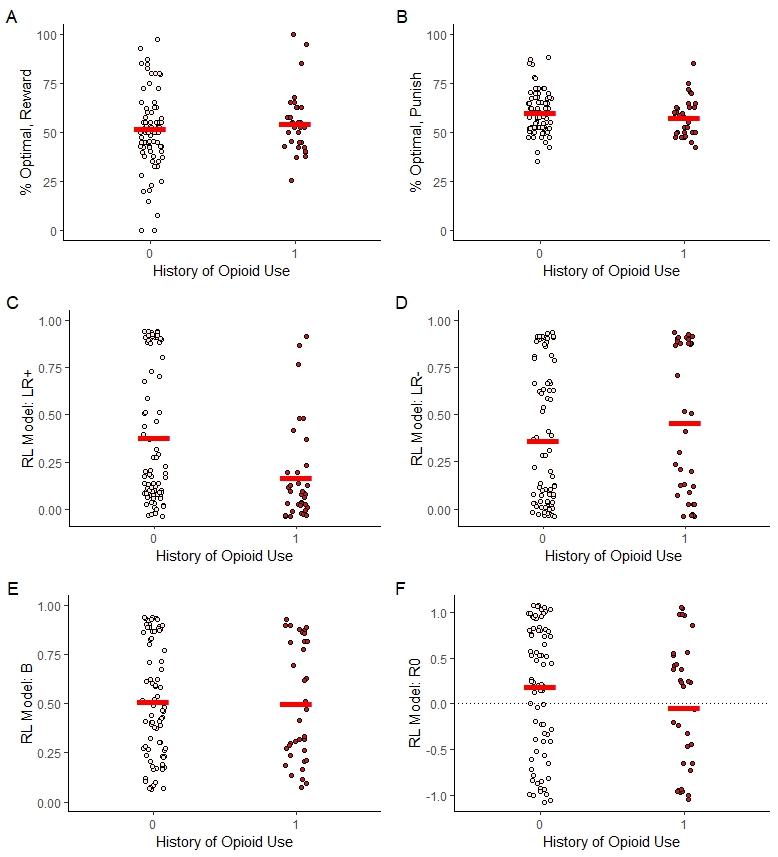 |
| --- |
| **Figure 12**. RPLT behavioral variables and RL model estimates, as a function of lifetime history of opioid use (1=yes, 0=no), including morphine, heroin, or use of prescription painkillers to get high. Other abbreviations/conventions as in Figures 2 & 3. |

#### 6.3.6. Supplementary analyses: Traumatic Brain Injury (TBI)

History of traumatic brain injury (TBI) is another variable that may affect behavior, including impaired reward sensitivity (21). To our knowledge, this has not been explicitly examined using the RPLT. Understanding of how TBI might contribute to predicting ASA is complicated in the current sample by the unequal distribution of TBI: as shown in Table 2, although 64% of the noSE group and most (11 of 13) in the OtherSE group had history of TBI, there were no cases of TBI history among the ASA group. Unfortunately, this hinders examination of TBI (since TBI emerges in GEE models as strongly predictive of noSE). This seems likely to be an issue with small sample size rather than a true finding that TBI is protective against upcoming ASA – particularly given that a majority of individuals in the study (both with and without TBI) had at least one prior ASA. Nevertheless, patients with a history of TBI showed slightly higher rates of optimal responding to reward-based stimuli, and larger values of *LR+*, compared to patients with no history of TBI (**Figure 13**).

| 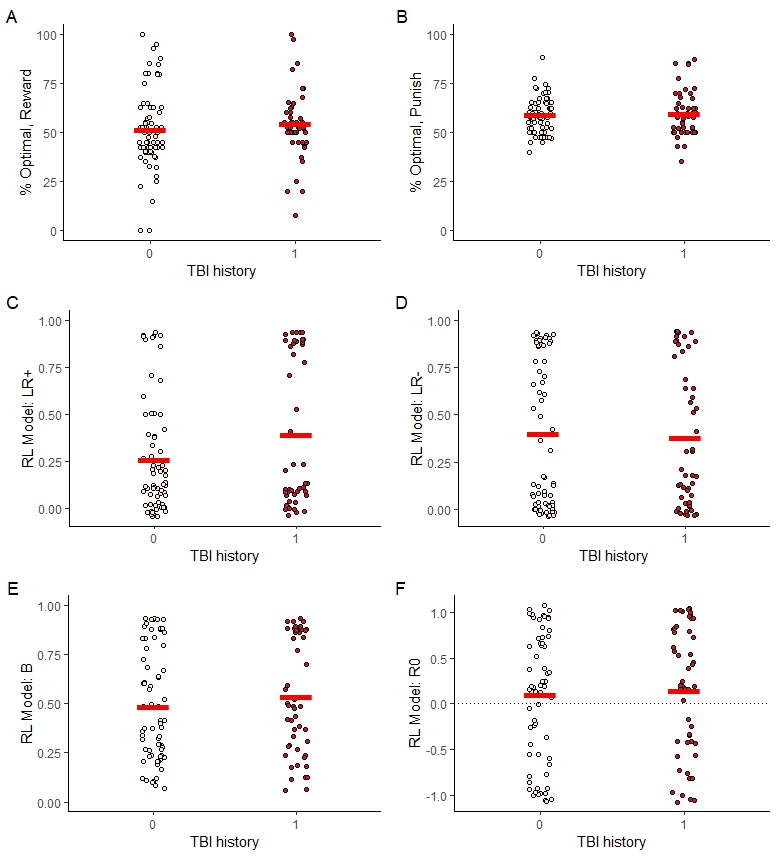 |
| --- |
| **Figure 13**. RPLT behavioral variables and RL model estimates, as a function of lifetime history of traumatic brain injury (TBI) (1=yes, 0=no). Other abbreviations/conventions as in Figures 2 & 3. |

## 6.4. Supplemental References

1. Posner K, Brown GK, Stanley B, Brent DA, Yershova K V, Oquendo MA, Currier GW, Glenn Melvin MA, Greenhill L, Shen S, et al. The Columbia-Suicide Severity Rating Scale: Initial validity and internal consistency findings from three multisite studies with adolescents and adults. *American Journal of Psychiatry* (2011) 168:1266–1277. www.cssrs.columbia.edu

2. Beck AT, Kovacs M, Weissman A. Assessment of suicidal intention: the Scale for Suicide Ideation. *J Consult Clin Psychol* (1979) 47:343–352. doi: 10.1037//0022-006x.47.2.343

3. Sheehan D V., Lecrubier Y, Sheehan KH, Amorim P, Janavs J, Weiller E, Hergueta T, Baker R, Dunbar GC. The Mini-International Neuropsychiatric Interview (M.I.N.I): The development and validation of a structured diagnostic psychiatric interview for DSM-IV and ICD-10. *Journal of Clinical Psychiatry* (1998) 59[suppl 20]:22–23.

4. Nasreddine ZS, Phillips NA, Bédirian V, Charbonneau S, Whitehead V, Collin I, Cummings JL, Chertkow H. The Montreal Cognitive Assessment, MoCA: A brief screening tool for mild cognitive impairment. *J Am Geriatr Soc* (2005) 53:695–699. doi: 10.1111/j.1532-5415.2005.53221.x

5. Waldron-Perrine B, Axelrod BN. Determining an appropriate cutting score for indication of impairment on the Montreal Cognitive Assessment. *Int J Geriatr Psychiatry* (2012) 27:1189–1194.

6. Schwab KA, Baker G, Ivins B, Sluss-Tiller M, Lux W, Warden D. The Brief Traumatic Brain Injury Screen (BTBIS): Investigating the validity of a self-report instrument for detecting traumatic brain injury in troops returning from deployment in Afghanistan and Iraq. *Neurology* (2006) 66:A235.

7. Brown GL, Goodwin FK, Ballenger JC, Goyer PF, Major LF. Aggression in humans correlates with cerebrospinal fluid amine metabolites. *Psychiatry Res* (1979) 1:131–139.

8. Lopez-Morinigo J-D, Boldrini M, Ricca V, Oquendo MA, Baca-Garcia E. Aggression, impulsivity and suicidal behavior in depressive disorders: A comparison study between New York City (USA), Madrid (Spain) and Florence (Italy). *J Clin Med* (2021) 10:3057.

9. Keane TM, Fairbank JA, Caddell JM, Zimering RT, Taylor KL, Mora CA. Clinical evaluation of a measure to assess combat exposure. *Psychological Assessment: A Journal of Consulting and Clinical Psychology* (1989) 1:53–55. doi: 10.1037/1040-3590.1.1.53

10. Beck AT, Steer RA, Ball R, Ranieri W. Comparison of Beck Depression Inventories -IA and -II in psychiatric outpatients. *J Pers Assess* (1996) 67:588–597.

11. Beck AT, Weissman A, Lester D, Trexler L. The measurement of pessimism: the hopelessness scale. *J Consult Clin Psychol* (1974) 42:861–865.

12. Moustafa AA, Gluck MA, Herzallah MM, Myers CE. The influence of trial order on learning from reward vs. punishment in a probabilistic categorization task: Experimental and computational analyses. *Front Behav Neurosci* (2015) 9:153. doi: 10.3389/fnbeh.2015.00153

13. Myers CE, Moustafa AA, Sheynin J, VanMeenen KM, Gilbertson MW, Orr SP, Beck KD, Pang KCH, Servatius RJ. Learning to Obtain Reward, but Not Avoid Punishment, Is Affected by Presence of PTSD Symptoms in Male Veterans: Empirical Data and Computational Model. *PLoS One* (2013) 8:e72508. doi: 10.1371/journal.pone.0072508

14. Frank MJ, Moustafa AA, Haughey HM, Curran T, Hutchison KE. Genetic triple dissociation reveals multiple roles for dopamine in reinforcement learning. *Proceedings of the National Academy of Sciences (USA)* (2007) 104:16311–16316. www.pnas.org/cgi/content/full/

15. Watkins CJCH, Dayan P. Q-Learning. *Mach Learn* (1992) 8:279–292.

16. Eddelbuettel D, Francois R. Rcpp: Seamless R and C++ Integration. *J Stat Softw* (2011) 40:1–18.

17. Akaike H. A new look at the statistical model identification. *IEEE Trans Autom Control* (1974) 19:716–723.

18. Herzallah MM, Moustafa AA, Natsheh JY, Abdellatif SM, Taha MB, Tayem YI, Sehwail MA, Amleh I, Petrides G, Myers CE, et al. Learning from negative feedback in patients with major depressive disorder is attenuated by SSRI antidepressants. *Front Integr Neurosci* (2013) 7:67. doi: 10.3389/fnint.2013.00067

19. Herzallah MM, Khdour HY, Taha AB, Elmashala AM, Mousa HN, Taha MB, Ghanim Z, Sehwail MM, Misk AJ, Balsdon T, et al. Depression Reduces Accuracy While Parkinsonism Slows Response Time for Processing Positive Feedback in Patients with Parkinson’s Disease with Comorbid Major Depressive Disorder Tested on a Probabilistic Category-Learning Task. *Front Psychiatry* (2017) 8:84. doi: 10.3389/fpsyt.2017.00084

20. Myers CE, Sheynin J, Balsdon T, Luzardo A, Beck KD, Hogarth L, Haber P, Moustafa AA. Probabilistic reward- and punishment-based learning in opioid addiction: Experimental and computational data. *Behavioural Brain Research* (2016) 296:240–248. doi: 10.1016/j.bbr.2015.09.018

21. Larson MJ, Kelly KG, Stigge-Kaufman DA, Schmalfuss IM, Perlstein WM. Reward context sensitivity impairment following severe TBI: an event-related potential investigation. *J Int Neuropsychol Soc* (2007) 13:615–25. doi: 10.1017/S1355617707070762
